# Exceptional concentration of fish diversity in Yasuní National Park, Ecuador (Napo River Basin)

**DOI:** 10.3897/BDJ.13.e136476

**Published:** 2025-03-13

**Authors:** Daniel Escobar-Camacho, Jonathan Valdiviezo-Rivera, Carolina Carrillo-Moreno, Pablo Argüello, Kelly Swing

**Affiliations:** 1 Instituto Biósfera, Universidad San Francisco de Quito, Quito, Ecuador Instituto Biósfera, Universidad San Francisco de Quito Quito Ecuador; 2 Instituto Nacional de Biodiversidad, Quito, Ecuador Instituto Nacional de Biodiversidad Quito Ecuador; 3 The Nature Conservancy, Puyo, Ecuador The Nature Conservancy Puyo Ecuador; 4 Departamento de Biología, Escuela Politécnica Nacional, Quito, Ecuador Departamento de Biología, Escuela Politécnica Nacional Quito Ecuador; 5 Tiputini Biodiversity Station, Universidad San Francisco de Quito, Quito, Ecuador Tiputini Biodiversity Station, Universidad San Francisco de Quito Quito Ecuador

**Keywords:** aquatic ecosystems, megadiversity, protected areas, teleosts, western Amazon

## Abstract

**Background:**

Despite limited access and rather deficient sampling in many lowland areas of eastern Ecuador, scientists have been able to demonstrate that this specific region of Amazonia houses extraordinarily high concentrations of species within several taxa – terrestrial and aquatic, plant and animal, vertebrate and invertebrate.

**New information:**

In this work, we developed an updated list of the ichthyofauna of the Yasuní National Park (YNP), based on an extensive literature review and databases of the most representative ichthyological collections from Ecuador. Our results yielded 458 species of freshwater fishes distributed in 47 families and 13 orders. This number exceeded previous fish lists from YNP and accounts for a considerable proportion of species inhabiting the Napo River Basin as well as the entire Amazon River Basin.

The higher-than-previously-reported species diversity within this protected area, the services these species provide to humans and the absence of invasive species underscore the need for greater efforts and investment in protecting and managing western Amazonian lands and waters.

## Introduction

Galacatos et al. 1996, Galacatos et al. 2004, Escobar-Camacho et al. 2015The Amazon River Basin is widely recognised as a region of extraordinary biological diversity, though popular discussions often focus primarily on its expansive lowlands. Within greater Amazonia, there are areas of exceptionally high species concentrations (from trees to insects and vertebrates) along a rather narrow crescent that extends from eastern Colombia in the north, through eastern Ecuador and eastern Peru and southwards to north-eastern Bolivia (Mittermeier et al. 2011, Kass et al. 2022, Sabatini 2022). The terrain along this strip of the western Amazon Basin exhibits striking topographic variation, with elevations ranging from over 6000 m at the Andean continental divide to less than 200 m within a few hundred kilometres (Sayre et al. 2008). This steep elevational gradient, combined with the equatorial position of Ecuador, has fostered the evolution and maintenance of hyper-diverse biota (Thomassen et al. 2011). The rapid shifting orographic feature of the young Andes has also driven biological diversification over the past few million years (Galvis et al. 2006, Wilkinson et al. 2010). Approximately half of the territory of Ecuador lies within the Amazonian biome, where protected areas, including Yasuní National Park (YNP), created in 1979, safeguard biodiversity and indigenous communities. YNP spans 1,029,566.32 hectares, with elevations ranging from 190 to 400 m a.s.l. (MAATE 2023).

**Figure 1.**
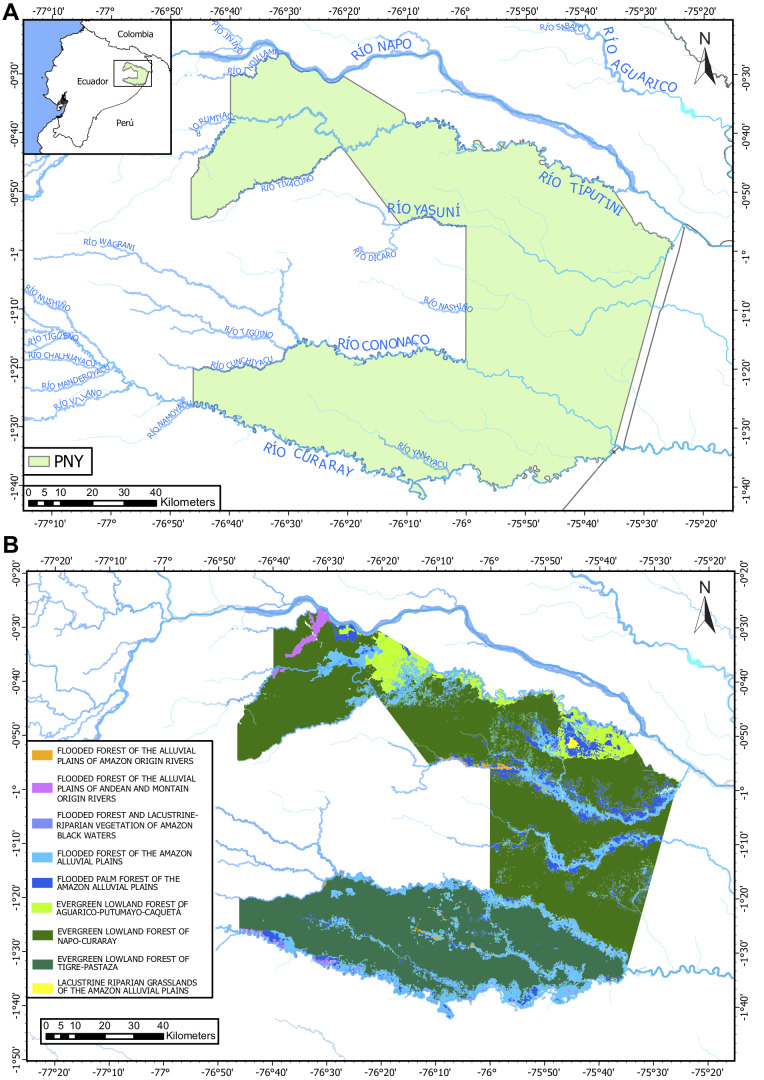
Map of Yasuní National Park showing its location (A) and ecosystems (B).

The unique location of YNP, where Andean rivers flow west to east, forms key tributaries of the upper Amazon. To the north, it is bounded by the Napo River and to the south by the Curaray River (Fig. 1), with a network of rivers (Tivacuno, Tiputini, Yasuní, Nashiño, Cononaco and Tigüino) of white and black waters (Galacatos et al. 2004, Galacatos et al. 1996, Escobar-Camacho et al. 2015). The YNP borders Peru to the east and the Waorani Ethnic Reserve to the west (Pappalardo et al. 2013). The Park´s transition from Andean piedmont to lowland floodplains support unique ecosystems, including Evergreen Lowland Forests, Várzea Forests of the Amazonian and Andean alluvial plains, Flooded Palm Forests and Lacustrine-Riparian Grasslands (MAATE 2023, Figs 1, 2). The YNP is home to three indigenous nationalities — Shuar, Kichwa and Waorani — and contains ancestral Waorani territory where two tribes live in voluntary isolation (Pappalardo et al. 2013, Trujillo Montalvo 2018). Critically, YNP boasts high biodiversity, particularly amongst freshwater fishes, which are one of the Park´s most diverse vertebrate groups (Bass et al. 2010).

Fish diversity studies in YNP began in the 1980s with ichthyological explorations of the Napo River Basin, led by the Field Museum of Chicago and Escuela Politécnica Nacional del Ecuador (Stewart et al. 1987, Ibarra and Stewart 1989). Over two decades, research documented 550 freshwater fish species in the Napo River Basin (Barriga 1994, Galacatos et al. 1996, Galacatos et al. 2004), including 253 species within YNP (Barriga 1994). Despite this progress, gaps remain in understanding the full extent of fish diversity, especially given ongoing taxonomic revisions (Valdiviezo-Rivera et al. 2020) and recent discoveries (Valdiviezo-Rivera et al. 2021, Chuctaya et al. 2023) in the region.

This study addresses these gaps by compiling an updated list of fish species occurring in YNP, informed by recent explorations, case studies and data from the two major ichthyological collections of Ecuador. By documenting the freshwater fish diversity of YNP, this work contributes to addressing the global freshwater biodiversity crisis (Reid et al. 2019, Tickner et al. 2020) by providing critical baseline data to provide information for conservation strategies and stakeholders.

## Materials and methods

To compile a list of fish species in YNP, an updated database was developed using previous datasets from the Napo Basin of Ecuador and Peru (Barriga 2012, Sanchez et al. 2013, Jézéquel et al. 2020). This database was curated, based on records within YNP boundaries and validated using the ichthyological collections of Ecuadorian museums: Museo de Historia Natural Gustavo Orcés – Escuela Politécnica Nacional (MEPN) and Colección Ictiológica del Instituto Nacional de Biodiversidad, Quito, Ecuador (MECN-DP). These records reflect decades of Amazonian expeditions and curatorial efforts. In addition, digital databases from the Field Museum of Natural History (FMNH) and the Smithsonian National Museum of Natural History (USNM), were verified for species absent in MEPN or MECN-DP. As it is possible that fish species registered outside YNP boundaries occur within the Reserve, for this study, we included records from a 15 km range beyond YNP limits. We did not include records from other protected areas nearby or more geographically distant sites despite any ecological similarities. Finally, species classification and ordering were also updated to current taxonomic revisions of each group according to Eschmeyer’s Catalog of Fishes – California Academy of Sciences.

## Data resources

Data underpinning this list have been gathered from records from three museum databases and literature. The museum data underpinning the reported list in this study are distributed across three sources: 1) the Darwin Core archive for MECN-DP can be accessed at https://bndb.sisbioecuador.bio/bndb/checklists/checklist.php?clid=15292&emode=0, 2) data from the Global Biodiversity Information Facility (GBIF) for MEPN are available at https://doi.org/10.60545/fl2c7w and 3) data of the fishes of Ecuador at the FMNH and USNM collection pages are available at https://collections-zoology.fieldmuseum.org/page/collections-data-fishes and https://collections.nmnh.si.edu/search/fishes/, respectively. The assembled data are available as a single file in the Dryad Data Repository (Escobar-Camacho 2024). This file includes voucher numbers for all species in the 'catalogNumber' column and the names of the museum collections in the 'collectionCode' column. In the checklist below, citations in 'Notes' indicate the literature reporting each species in the YNP area.

## Checklists

### Fish Species Checklist of Yasuni National Park

#### Myliobatiformes

74EEC9D8-ADAC-5BC7-B683-DE87F192B29D

#### Potamotrygonidae

C10D8AFF-794E-57F3-A77D-0C8B3AC986A8

#### Plesiotrygon iwamae

Rosa, Castello & Thorson 1987

5BDF1079-463E-55D8-BE7B-2E5F2657B578

##### Distribution

Ecuador, Peru, Brazil.

##### Notes

Barriga (1994).

#### Potamotrygon motoro

(Müller & Henle 1841)

95D88A14-D114-5767-A68E-891C4B49B23B

##### Distribution

tropical S.A., east of Andes.

##### Notes

Barriga (1994), Galacatos et al. (2004), Valdiviezo-Rivera et al. (2018), Jácome-Negrete et al. (2018).

#### Potamotrygon scobina

Garman 1913

DC86E3A1-51E0-5B45-9526-4FEB9C2EE90B

##### Distribution

Amazonia.

#### Osteoglossiformes

33D585F2-40EF-59BD-BDAE-4146044A05A6

#### Osteoglossidae

A04C549F-EF1F-5AA5-8C87-00F100656AB9

#### Osteoglossum bicirrhosum

(Cuvier 1829)

6FBC1F97-45A2-557C-80A7-DDED9A1385C5

##### Distribution

Amazonia.

##### Notes

Barriga (1994), Galacatos et al. (2004), Jácome-Negrete (2013).

#### Arapaimidae

9D0EEB27-D351-5F19-8314-59A6A191D577

#### Arapaima cf. gigas f. cf.

DA9AF63D-560E-5C1D-8710-C502BE45B688

##### Distribution

Amazonia.

##### Notes

Barriga (1994), WCS (2007), Jácome-Negrete (2013).

#### Clupeiformes

A9D28D4F-0AE6-5D64-9551-0571E9ADEDE7

#### Engraulidae

7D0E1019-D8C2-51BD-9ED1-4C1403729EC5

#### Anchoviella alleni

(Myers 1940)

6B29A865-9CA4-58D2-93C2-3001FE1BBD9D

##### Distribution

Western Amazonia.

##### Notes

Barriga (1994), Valdiviezo-Rivera et al. (2018).

#### Anchoviella sp. 1

A2B6839A-0896-519C-893B-98463CEC3B15

##### Distribution

Eastern Ecuador, Limoncocha Lagoon.

#### Lycengraulis batesii

(Günther 1868)

66B619B6-8172-5C54-8A23-1CDC85501A75

##### Distribution

Amazonia.

##### Notes

Barriga (1994), Galacatos et al. (2004).

#### Pristigasteridae

032E037F-36F3-56AD-8924-713043266935

#### Ilisha amazonica

(Miranda Ribeiro 1920)

0979F7AD-63D1-5DC0-952D-3F9BE136DC17

##### Distribution

Amazonia.

##### Notes

Galacatos et al. (2004).

#### Pellona castelnaeana

Valenciennes 1847

F1B870A4-69BF-5AAB-99A2-EFB6C57DD09E

##### Distribution

Colombia, Ecuador, Peru, Bolivia, Brazil.

##### Notes

Galacatos et al. (2004), Jácome-Negrete (2013), Jácome-Negrete et al. (2018).

#### Pristigaster cayana

Cuvier 1829

F2118851-E142-5318-AF5C-5A8D91910EB0

##### Distribution

Colombia, Ecuador, Peru, Brazil.

##### Notes

Barriga (1994), Jácome-Negrete (2013), Jácome-Negrete et al. (2018).

#### Pristigaster whiteheadi

Menezes & de Pinna 2000

CDF38AD0-7F00-5990-AC57-F593408E5EB5

##### Distribution

Colombia, Ecuador, Peru, Brazil.

##### Notes

Galacatos et al. (2004).

#### Characiformes

6CDA8768-5912-5893-A53A-8166426DCDB4

#### Crenuchidae

38F2DEB5-7222-5FF0-A47C-23C98F5518AC

#### Characidium boehlkei

Géry 1972

C01EB9BA-0876-564A-91B0-8BB2622092E8

##### Distribution

Napo River Basin, Ecuador.

##### Notes

Barriga (1994).

#### Characidium etheostoma

Cope 1872

D00361B1-ED9E-594D-A77C-C308ADBFC279

##### Distribution

Amazonia.

##### Notes

Tobes et al. (2022).

#### Characidium geryi

(Zarske 1997)

39850B9C-A200-59EB-81FB-2155E73C68B2

##### Distribution

Peru, Ecuador.

#### Characidium purpuratum

Steindachner 1882

5DD0D82C-A3C6-5269-9D64-096769A4DDB2

##### Distribution

Tributaries of the upper Amazon in Bolivia, Ecuador and Peru.

#### Characidium steindachneri

Cope 1878

FC0A9031-E946-5306-86E3-DF184947FDD8

##### Distribution

Amazonia, Guyanas.

##### Notes

Tobes et al. (2022).

#### Characidium sp. 1

A4E76590-F21D-5729-A55B-5EA24BF1A71F

##### Distribution

Eastern Ecuador, Napo River, Yasuní River.

##### Notes

Stewart et al. 2002, Valdiviezo-Rivera et al. (2018).

#### Elachocharax pulcher

Myers 1927

658517CA-6FE5-5189-88E8-BF6E412AF4A8

##### Distribution

Amazonia, upper Orinoco.

#### Melanocharacidium rex

(Böhlke 1958)

5294CBD2-F1C0-5FF9-9ADE-D8F8EC3BA1C5

##### Distribution

South-eastern Ecuador.

#### Microcharacidium eleotrioides

(Géry 1960)

F7B8A224-E96E-532A-AEB0-2537F40F9C91

##### Distribution

Coastal streams (French Guiana, Guyana, Suriname, Bolivia, Brazil, Ecuador and Colombia).

#### Odontocharacidium aphanes

(Weitzman & Kanazawa 1977)

5449F87D-E42C-5FE0-B20F-F5CBC2777618

##### Distribution

Mostly western central Amazonia.

#### Erythrinidae

4281BCA1-79CF-5B44-B4DF-69CB5DE5E2E3

#### Erythrinus erythrinus

(Bloch & Schneider 1801)

9E817E6D-CCF8-53DE-BC9D-E9C8B5DE5744

##### Distribution

All Amazonia, plus northern drainages.

##### Notes

Barriga (1994), Galacatos et al. (2004).

#### Hoplerythrinus unitaeniatus

(Spix & Agassiz 1829)

A6F16815-B700-5FC1-8EA6-52C750BEF4A6

##### Distribution

All Amazonia, plus northern drainages.

##### Notes

Barriga (1994), Galacatos et al. (2004).

#### Hoplias malabaricus

(Bloch 1794)

DC566EBE-394A-5A45-8BB9-9CF31139CF71

##### Distribution

Widespread Neotropical.

##### Notes

Barriga (1994), Galacatos et al. (2004), WCS (2007), Jácome-Negrete (2013), Jácome-Negrete et al. (2018), Tobes et al. (2022).

#### Parodontidae

78DDE74F-8009-5247-9F1D-C0C220A2F971

#### Parodon buckleyi

Boulenger 1887

CDCC1A26-324E-5B44-8620-07441CB33A9E

##### Distribution

Widespread Amazonia.

##### Notes

Barriga (1994), Tobes et al. (2022).

#### Parodon pongoensis

(Allen 1942)

D0FE355F-6293-599B-A321-6A33BFDFC1E0

##### Distribution

Western Amazonia.

##### Notes

Barriga (1994), Escobar-Camacho et al. (2015).

#### Cynodontidae

2A9EF1E7-2F12-5974-BD43-869D30C8058B

#### Cynodon gibbus

(Spix & Agassiz 1829)

E5578423-9850-5CA3-B87E-780F41E96EE8

##### Distribution

All Amazonia, plus northern drainages.

##### Notes

Barriga (1994), Galacatos et al. (2004).

#### Hydrolycus scomberoides

(Cuvier 1819)

191A6BE3-E906-53BB-89F5-20A012C7021F

##### Distribution

All Amazonia, Orinoco.

##### Notes

Barriga (1994), Galacatos et al. (2004), Jácome-Negrete (2013), Jácome-Negrete et al. (2018).

#### Rhaphiodon vulpinus

Spix & Agassiz 1829

C48E285B-C348-5348-B5E0-422903921B51

##### Distribution

All Amazonia, Orinoco.

##### Notes

Barriga (1994), Galacatos et al. (2004), Jácome-Negrete (2013), Jácome-Negrete et al. (2018).

#### Serrasalmidae

7A480F5C-7CB7-5C29-832F-0EC3343C378B

#### Colossoma macropomum

(Cuvier 1816)

76D42B58-3B25-5F4E-A72D-26D0C68B6EC7

##### Distribution

All Amazonia, plus northern drainages.

##### Notes

Barriga (1994) Galacatos et al. (2004), WCS (2007), Jácome-Negrete (2013).

#### Myleus pacu

(Jardine 1841)

6AF883DA-50AE-510C-8985-E47FA75585C2

##### Distribution

All Amazonia, plus northern drainages.

##### Notes

Ibarra and Stewart 1989, Galacatos et al. 1996.

#### Mylossoma albiscopum

(Cope 1872)

280F92E4-D3F1-51D4-9619-BB92109699DC

##### Distribution

Widespread Amazonia, Orinoco.

##### Notes

Barriga (1994), Galacatos et al. (2004), Valdiviezo-Rivera et al. (2020).

#### Mylossoma aureum

(Spix & Agassiz 1829)

BFB5AEA3-81DA-5110-BAF2-9D31B655910A

##### Distribution

All Amazonia, plus northern drainages.

##### Notes

Barriga (1994), Galacatos et al. (2004), Barriga (2011), Guarderas and Jácome-Negrete (2013), Valdiviezo-Rivera et al. (2020).

#### Pygocentrus nattereri

Kner 1858

AAE60E4B-1B7C-5AFB-9C28-774188CFE89F

##### Distribution

Widespread Amazonia.

##### Notes

Barriga (1994), Galacatos et al. (2004), Jácome-Negrete 2013, Escobar-Camacho et al. (2015), Valdiviezo-Rivera et al. (2018), Jácome-Negrete et al. (2018).

#### Serrasalmus elongatus

Kner 1858

C227B513-A5C0-521F-936B-25EE47BBED51

##### Distribution

Widespread Amazonia, Orinoco.

##### Notes

Galacatos et al. (2004).

#### Serrasalmus rhombeus

(Linnaeus 1766)

42B29373-6268-540E-89C6-4CF0205DDF92

##### Distribution

Widespread Amazonia, plus northern drainages.

##### Notes

Barriga (1994), Galacatos et al. (2004), Jácome-Negrete (2013), Escobar-Camacho et al. (2015), Valdiviezo-Rivera et al. (2018), Jácome-Negrete et al. (2018)Jácome-Negrete et al. 2018.

#### Hemiodontidae

385AA4C7-380C-5A2F-B6B4-556BE5647EF7

#### Anodus elongatus

Agassiz 1829

BD392989-B4C9-50CB-9042-4C29EBC4B9F0

##### Distribution

Amazonia, Orinoco.

##### Notes

Barriga (1994), Galacatos et al. (2004), Jácome-Negrete (2013), Jácome-Negrete et al. (2018).

#### Hemiodus microlepis

Kner 1858

CFFBB9F1-01C7-5268-BE02-B902D5DA54C1

##### Distribution

Amazonia, Orinoco.

##### Notes

Jácome-Negrete (2013), Jácome-Negrete et al. (2018).

#### Hemiodus unimaculatus

(Bloch 1794)

5350C7C2-ED2F-577C-86AD-EBFC7D02BE81

##### Distribution

Widespread Amazonia, Orinoco.

##### Notes

Barriga (1994), Galacatos et al. (2004).

#### Anostomidae

F39111C7-6529-5596-B701-1E704B42A224

#### Abramites hypselonotus

(Günther 1868)

D936C502-1F3A-5C7C-AA61-F8D2D2059DBC

##### Distribution

Widespread Amazonia, Orinoco.

##### Notes

Barriga (1994).

#### Laemolyta garmani

(Borodin 1931)

2D502023-993C-50AA-8273-92139E7EC2FD

##### Distribution

Widespread Amazonia.

##### Notes

Barriga (1994), Galacatos et al. (2004).

#### Leporellus vittatus

(Valenciennes 1850)

BF9CC76C-C42A-571B-A639-D66C6A1CBB8C

##### Distribution

Widespread Amazonia, Orinoco.

##### Notes

Barriga (1994).

#### Leporinus agassizii

Steindachner 1876

99B46E65-4080-566C-8BD2-3F3945820289

##### Distribution

Amazonia.

##### Notes

Barriga (1994), Galacatos et al. (2004), Jácome-Negrete (2013), Jácome-Negrete et al. (2018).

#### Leporinus cf. fasciatus

E40170F6-5E42-56C8-848B-6A9307CEDF1A

##### Distribution

Amazonia.

##### Notes

Jácome-Negrete (2013), Jácome-Negrete et al. (2018).

#### Leporinus friderici

(Bloch 1794)

2CA41B17-82DD-575D-922B-8054085A5E61

##### Distribution

Widespread Amazonia, Orinoco.

##### Notes

Barriga (1994), Jácome-Negrete (2013), Valdiviezo-Rivera et al. (2018), Jácome-Negrete et al. (2018).

#### Leporinus sp. 1

0B51EFB2-4F0A-54F6-A3A8-F81AFB3F6C28

##### Distribution

Manduro and Samona Rivers, right bank of the Napo River, one hour downstream from Francisco de Orellana.

#### Leporinus jatuncochi

Ovchynnyk 1971

0566FF27-BBB6-524E-AEEF-5A7DABB7FBC6

##### Distribution

Napo Basin, Ecuador.

##### Notes

Barriga (1994).

#### Leporinus niceforoi

Fowler 1943

1C67F7E1-7B1D-56AB-A5F8-BDFDA141B0FA

##### Distribution

Amazonian Colombia, Ecuador.

##### Notes

Barriga (1994), Galacatos et al. (2004).

#### Leporinus pearsoni

Fowler 1940

D91DDD05-A13C-55DE-AED4-043596054082

##### Distribution

Western Amazonia.

##### Notes

Barriga (1994).

#### Leporinus cf. striatus

59EEA75B-0255-5EF9-9CEC-77FA3AB8D007

##### Distribution

Widespread Amazonia.

##### Notes

Barriga (1994).

#### Leporinus subniger

Fowler 1943

DEC8FE5D-DAE7-5456-8F98-F07366B5339B

##### Distribution

Amazonian Colombia, Ecuador.

#### Megaleporinus cf. trifasciatus

480F308B-6A3C-5128-A129-138FB964FE59

##### Distribution

Amazonia.

##### Notes

Barriga (1994), Galacatos et al. (2004).

#### Pseudanos trimaculatus

(Kner 1858)

ECA0242F-0853-5404-A581-28D1D13EA3D5

##### Distribution

Amazonia, plus northern drainages.

##### Notes

Barriga (1994).

#### Schizodon fasciatus

Spix & Agassiz 1829

E6A35B70-7840-5E35-877E-14F2B7B886B2

##### Distribution

Widespread Amazonia, plus northern drainages.

##### Notes

Barriga (1994), Galacatos et al. (2004), Jácome-Negrete (2013), Jácome-Negrete et al. (2018).

#### Chilodontidae

F0DC1740-8B37-5380-925E-1CA1BBC5A0AF

#### Caenotropus labyrinthicus

(Kner 1858)

1A7ABDB4-8C09-5EC2-AE18-4E07A08E0C93

##### Distribution

Widespread Amazonia, plus northern drainages.

##### Notes

Barriga (1994), Galacatos et al. (2004), Jácome-Negrete (2013), Jácome-Negrete et al. (2018).

#### Chilodus punctatus

Müller & Troschel 1844

BBC3FD6B-77D4-56EF-8456-220F491E7111

##### Distribution

Widespread Amazonia, plus northern drainages.

##### Notes

Barriga (1994), Galacatos et al. (2004).

#### Curimatidae

C699F607-BD04-575B-987C-8711E59A5E06

#### Curimata aspera

Günther 1868

5775071D-47FB-5B90-BE80-3D11871C19CD

##### Distribution

Western Amazonia.

##### Notes

Barriga (1994), Galacatos et al. (2004), Valdiviezo-Rivera et al. (2018).

#### Curimata cisandina

(Allen 1942)

905E2C57-1D1E-5450-B14B-0FD031CDB295

##### Distribution

Western Amazonia.

##### Notes

Barriga (1994), Galacatos et al. (2004).

#### Curimata knerii

Steindachner 1876

DBB16207-7B0C-50E9-9D0C-F4FF672768E8

##### Distribution

Middle, upper Amazon.

##### Notes

Barriga (1994).

#### Curimata roseni

Vari 1989

3255D910-2360-504A-9462-45092F18823F

##### Distribution

Amazonia, upper Orinoco.

##### Notes

Galacatos et al. (2004), Valdiviezo-Rivera et al. (2018).

#### Curimata vittata

(Kner 1858)

F2977992-3C43-5E62-B6FC-7034FF6A927E

##### Distribution

Amazonia, upper Orinoco.

##### Notes

Barriga (1994), Galacatos et al. (2004), Jácome-Negrete (2013), Jácome-Negrete et al. (2018).

#### Curimatella alburnus

(Müller & Troschel 1844)

40FA4604-A3DC-5E71-B524-5EA450F558ED

##### Distribution

Widespread Amazonia.

##### Notes

Barriga (1994), Galacatos et al. (2004).

#### Curimatella meyeri

(Steindachner 1882)

3B6523B0-2541-5A0E-9138-1A0E8152215D

##### Distribution

Middle, upper Amazon.

##### Notes

Galacatos et al. (2004)

#### Curimatopsis macrolepis

(Steindachner 1876)

D5FB805B-D2B2-55A6-8D07-7359E85E0CE9

##### Distribution

Amazon, Orinoco.

##### Notes

Galacatos et al. (2004).

#### Curimatopsis microlepis

Eigenmann & Eigenmann 1889

8A730C45-CBAC-54D2-8F50-85B4465E340E

##### Distribution

Amazonia.

#### Cyphocharax gouldingi

Vari 1992

A75E31CB-D47F-5DFC-8379-47B0677F4D29

##### Distribution

Brazil, eastern Ecuador.

#### Cyphocharax laticlavius

Vari & Blackledge 1996

38F8DEFD-CD61-5FF5-B96F-53C6285085DA

##### Distribution

Napo, Ecuador.

##### Notes

Galacatos et al. (2004).

#### Cyphocharax notatus

(Steindachner 1908)

C439E8AC-534B-597B-B411-055F9AF89681

##### Distribution

Amazonia.

#### Cyphocharax pantostictos

Vari & Barriga Salazar 1990

C698F1B4-C385-5578-AFBE-12C7804DBAB2

##### Distribution

Western Amazonia.

##### Notes

Barriga (1994).

#### Cyphocharax plumbeus

(Eigenmann & Eigenmann 1889)

00E633A1-1A2F-532C-9936-10CA2E6E5DEA

##### Distribution

Amazonia.

##### Notes

Galacatos et al. (2004).

#### Cyphocharax spiluropsis

(Eigenmann & Eigenmann 1889)

0FE138AE-5F15-5300-8B0E-B91BA69CE5A4

##### Distribution

Central, western Amazonia.

##### Notes

Galacatos et al. (2004).

#### Cyphocharax vexillapinnus

Vari 1992

0D479F51-E649-5E3D-A26F-BC348C098463

##### Distribution

Central, western Amazonia.

#### Potamorhina altamazonica

(Cope 1878)

08AECFDA-196F-5CCF-975C-8EF12F3E9821

##### Distribution

Amazon, Orinoco.

##### Notes

Barriga (1994), Galacatos et al. (2004), Valdiviezo-Rivera et al. (2018).

#### Potamorhina latior

(Spix & Agassiz 1829)

E477F103-BDCF-571E-9756-598F4897DE60

##### Distribution

Amazonia.

##### Notes

Barriga (1994), Galacatos et al. (2004), Jácome-Negrete (2013), Jácome-Negrete et al. (2018).

#### Psectrogaster amazonica

Eigenmann & Eigenmann 1889

2F7E98FF-F7F3-5AC6-B6A2-42A31C42508D

##### Distribution

Amazonia.

##### Notes

Barriga (1994), Galacatos et al. (2004), Jácome-Negrete (2013), Jácome-Negrete et al. (2018).

#### Psectrogaster rutiloides

(Kner 1858)

49C38D1C-CF3B-54B4-A8B6-1796420381A6

##### Distribution

Amazonia.

##### Notes

Galacatos et al. (2004).

#### Steindachnerina bimaculata

(Steindachner 1876)

30FF73D6-A9D1-5237-A869-C66B4A257779

##### Distribution

Amazon, Orinoco.

##### Notes

Barriga (1994), Galacatos et al. (2004), Valdiviezo-Rivera et al. (2018), Jácome-Negrete et al. (2018).

#### Steindachnerina dobula

(Günther 1868)

4AFF1185-BCE0-5942-B72E-D7D109B120A3

##### Distribution

Western Amazonia.

##### Notes

Barriga (1994), Tobes et al. (2022).

#### Steindachnerina guentheri

(Eigenmann & Eigenmann 1889)

32191E10-9977-54D9-BB3E-DE62A62459F6

##### Distribution

Western Amazonia, northern drainages.

#### Steindachnerina leuciscus

(Günther 1868)

5B615B4C-F0A1-559C-B196-C26021BE5372

##### Distribution

Amazonia.

#### Steindachnerina planiventris

Vari & Williams Vari 1989

FB7B29B1-2F70-5F27-8D8E-E93074F40160

##### Distribution

Central, western Amazonia.

#### Prochilodontidae

D5FBF689-6821-5F7E-A2A2-9D0F9E8FA237

#### Prochilodus nigricans

Spix & Agassiz 1829

3FDE034D-3B41-56C5-A24D-75200B670A71

##### Distribution

Widespread Amazonia.

##### Notes

Barriga (1994), Galacatos et al. (2004), WCS (2007), Jácome-Negrete (2013), Escobar-Camacho et al. (2015), Jácome-Negrete et al. (2018).

#### Semaprochilodus insignis

(Jardine 1841)

9579DD96-DDCE-578F-AF48-04FDC4F11999

##### Distribution

Central, western Amazonia.

##### Notes

Barriga (1994).

#### Lebiasinidae

38DF3CF8-D82E-5771-8EA1-C3C4C7B43943

#### Copeina guttata

(Steindachner 1876)

EBB1388D-5E2A-537C-9D6B-BB79331D5C8E

##### Distribution

Central, western Amazonia.

##### Notes

Barriga (1994).

#### Lebiasina elongata

(Boulenger 1887)

9EA85779-1970-5000-B047-AE97AECABD7C

##### Distribution

Eastern Ecuador and Peru.

##### Notes

Barriga (1994).

#### Nannostomus eques

Steindachner 1876

6EA08662-563A-5F99-9D0B-B4904F384DE5

##### Distribution

Middle, upper Amazon.

##### Notes

Barriga (1994).

#### Nannostomus marginatus

Eigenmann 1909

F4CCFCD3-2240-591F-AB0F-9932B6AE0C6D

##### Distribution

Widespread Amazonia.

##### Notes

Barriga (1994), Barriga (2011).

#### Pyrrhulina brevis

Steindachner 1876

8F262A79-AE39-553B-BC8D-90D8C68EA101

##### Distribution

Amazonia.

#### Pyrrhulina eleanorae

Fowler 1940

72576C58-0335-5412-AC3F-3E5D339F43C1

##### Distribution

Upper Amazon.

#### Pyrrhulina obermulleri

Myers 1926

59D7C1B3-14D2-50C4-81C4-624963DA6871

##### Distribution

Upper Amazon.

#### Pyrrhulina semifasciata

Steindachner 1876

E83A2A1B-C083-5E34-B683-B95406E9765C

##### Distribution

Amazonia.

##### Notes

Barriga (1994).

#### Pyrrhulina zigzag

Zarske & Géry 1997

772123EC-D787-516E-8CE0-6511C92A97F6

##### Distribution

Upper Amazon.

#### Ctenoluciidae

04DE398E-A2FB-5782-84BF-E58A8E4BE25B

#### Boulengerella cuvieri

(Spix & Agassiz 1829)

73D4892D-E431-56FB-8E54-3AD8DE4CAEDD

##### Distribution

Amazonia, plus northern drainages.

##### Notes

Galacatos et al. (2004).

#### Boulengerella maculata

(Valenciennes 1850)

9FAFBFF6-8E35-5488-8D99-1C7D837CF99E

##### Distribution

Amazon, Orinoco.

##### Notes

Barriga (1994), Galacatos et al. (2004), Jácome-Negrete (2013), Jácome-Negrete et al. (2018).

#### Triportheidae

34F494AD-1413-5B50-9B3B-5692911E0981

#### Engraulisoma taeniatum

Castro 1981

F06ECC89-BFAB-508C-A1BD-ED1B6C69FF86

##### Distribution

Napo Basin, upper Paraguay.

#### Triportheus albus

Cope 1872

C8FB1586-C5E4-5F27-B931-19349028A602

##### Distribution

Widespread Amazonia.

##### Notes

Barriga (1994), Galacatos et al. (2004), Jácome-Negrete (2013), Jácome-Negrete et al. (2018).

#### Triportheus angulatus

(Spix & Agassiz 1829)

5B5C9504-05A1-58E2-96E4-5127A8E7FAB2

##### Distribution

Widespread Amazonia.

##### Notes

Jácome-Negrete (2013), Jácome-Negrete et al. (2018).

#### Triportheus auritus

(Valenciennes 1850)

99093C8D-0800-54A1-A6F4-93C78DC3991C

##### Distribution

Widespread Amazonia.

##### Notes

Escobar-Camacho et al. (2015).

#### Triportheus pictus

(Garman 1890)

A493B66F-5CD4-5FCE-BCBF-83B01D0C5E76

##### Distribution

Widespread Amazonia.

##### Notes

Galacatos et al. (2004).

#### Gasteropelecidae

44097939-02AE-55F5-84DE-A1852823AAC2

#### Carnegiella myersi

Fernández-Yépez 1950

6C80D864-78F3-5A83-A25E-08B2AEF3E902

##### Distribution

Western Amazonia.

#### Carnegiella schereri

Fernández-Yépez 1950

735F19C6-69C1-575A-B79F-B23363932C78

##### Distribution

Brazil, western Amazonia.

##### Notes

Barriga (1994), Galacatos et al. (2004).

#### Carnegiella strigata

(Günther 1864)

146227B3-F052-5D6A-9F97-7EF5DAE2E134

##### Distribution

Widespread Amazonia.

##### Notes

Barriga (1994), Galacatos et al. (2004).

#### Gasteropelecus sternicla

(Linnaeus 1758)

3D51B5D5-8975-5086-A3C4-D1578C9DE8CD

##### Distribution

Upper/middle Amazonia, Guianas, Paraguay Basin.

##### Notes

Barriga (1994).

#### Thoracocharax securis

(De Filippi 1853)

B4C4A8BE-FE52-54A1-A7FE-CB6E9D1C66C3

##### Distribution

Upper/middle Amazonia, Guianas, Paraguay Basin.

##### Notes

Barriga (1994), Galacatos et al. (2004).

#### Thoracocharax stellatus

(Kner 1858)

17F27295-B984-532F-A109-A8A7C1C8671A

##### Distribution

Amazon, Orinoco, Paraná,

##### Notes

Barriga (1994), Galacatos et al. (2004), Jácome-Negrete (2013), Escobar-Camacho et al. (2015), Jácome-Negrete et al. (2018).

#### Bryconidae

C1DBC8CA-ADCF-53B4-A91A-EBB8E5163A95

#### Brycon coxeyi

Fowler 1943

B60C7E8B-DFAE-5E89-A7E5-C992512EA6A1

##### Distribution

Eastern Ecuador.

#### Brycon melanopterus

(Cope 1872)

245AB1BF-FF44-5BE4-9760-DED435C8FAA6

##### Distribution

Amazonia.

##### Notes

Barriga (1994), WCS (2007).

#### Brycon pesu

Müller & Troschel 1845

07D3336A-6C83-5026-98E7-150F05CE7EBB

##### Distribution

Amazonia, plus northern drainages.

#### Salminus hilarii

Valenciennes 1850

D0B63F49-A4C7-5CDC-A296-AEECF7261983

##### Distribution

Western and south-eastern Amazonia.

##### Notes

Galacatos et al. (2004)

#### Iguanodectidae

A996742E-8CF5-541B-817D-E29228A4DA04

#### Bryconops alburnoides

Kner 1858

1BF124D9-2123-5E22-B203-0404D8A57317

##### Distribution

Amazonia, Orinoco.

#### Bryconops caudomaculatus

(Günther 1864)

D96EFCC6-D9E0-5753-9BF1-06BC8845F435

##### Distribution

Amazonia, plus northern drainages.

#### Iguanodectes spilurus

(Günther 1864)

8D7B81F8-D588-58C4-A7D4-C96A6B5CC4C1

##### Distribution

Amazonia, Orinoco.

##### Notes

Escobar-Camacho et al. (2015).

#### Piabucus melanostoma

Holmberg 1891

C5659BCA-1A50-58F8-A867-893A4971DE8A

##### Distribution

South-western Amazonia, Paraguay Basin.

#### Acestrorhynchidae

45009907-6392-5FC0-9F31-485F879734A5

#### Acestrorhynchus abbreviatus

(Cope 1878)

135C7DD4-0F50-53F8-A759-C596AC3814A6

##### Distribution

Western and south-western Amazonia.

##### Notes

Galacatos et al. (2004).

#### Acestrorhynchus falcatus

(Bloch 1794)

441CFA6A-F2F3-5551-B470-34B5A841ACE1

##### Distribution

Amazonia, plus northern drainages.

##### Notes

Barriga (1994), Galacatos et al. (2004).

#### Acestrorhynchus falcirostris

(Cuvier 1819)

6F9A3F58-6EF6-578C-9A1B-31E8378BF5B0

##### Distribution

Amazonia, plus northern drainages.

##### Notes

Galacatos et al. (2004).

#### Acestrorhynchus microlepis

(Jardine 1841)

AC6C6A7E-014A-5292-8BF9-376E477118C5

##### Distribution

Amazonia, plus northern drainages.

##### Notes

Barriga (1994), Galacatos et al. (2004).

#### Gnathocharax steindachneri

Fowler 1913

CEC56F85-9B57-5859-969E-1408702B0637

##### Distribution

Amazonia, Orinoco.

#### Stevardiidae

A90AA585-6F0B-586B-8041-2BB8E5F14B2F

#### Boehlkea orcesi

(Böhlke 1958)

83990E20-7526-5026-9C61-1BE44D511906

##### Distribution

Eastern Ecuador.

#### Bryconacidnus ellisae

(Pearson 1924)

68724D37-EE6E-5905-9984-A7F7878204A0

##### Distribution

Upper Amazon.

##### Notes

Barriga (1994).

#### Bryconacidnus paipayensis

(Pearson 1929)

354AB9D1-37AD-5FC0-877D-7C9D33A2037A

##### Distribution

Upper Amazon.

#### Chrysobrycon hesperus

(Böhlke 1958)

7B4C8F43-309A-5659-A065-BACBBB6B3894

##### Distribution

Napo River Basin.

##### Notes

Valdiviezo-Rivera et al. (2018).

#### Creagrutus amoenus

Fowler 1943

0B140735-54F2-5A8B-9FC5-235B865E060C

##### Distribution

Wastern Ecuador, south-eastern Colombia.

##### Notes

Tobes et al. (2022).

#### Creagrutus barrigai

Vari & Harold 2001

4FD89D4E-B029-5867-A381-66C4222745C5

##### Distribution

Eastern Ecuador, north-eastern Peru.

#### Creagrutus cochui

Géry 1964

9461EE7C-D60B-5122-8793-6847FF6678AF

##### Distribution

Western Amazonia.

#### Creagrutus flavescens

Vari & Harold 2001

9D8C989B-A9E5-58EE-B5F3-2E21FF750C97

##### Distribution

Andean piedmont, western Amazonia.

#### Creagrutus gephyrus

Böhlke & Saul 1975

CBD88B55-5E16-55A5-8FF8-6295FB00C7FF

##### Distribution

Eastern Ecuador, north-eastern Peru.

##### Notes

Barriga (1994).

#### Creagrutus gracilis

Vari & Harold 2001

954CB5A9-F0FC-5076-9860-4BFE912A279E

##### Distribution

Eastern Ecuador, north-eastern Peru.

#### Creagrutus sp. 1

8A10BD16-D158-5783-8FB3-23FA7BD35862

##### Distribution

Eastern Ecuador, Sunka stream, tributary of Tiputini River.

##### Notes

Valdiviezo-Rivera et al. (2018).

#### Creagrutus sp. 2

9CF85C20-E6BF-5DDE-BE14-16E9ADA54905

##### Distribution

Eastern Ecuador, unnamed stream tributary of Divaro River.

#### Gephyrocharax major

Myers 1929

37314C00-156F-54A5-83D0-C2F68C0913ED

##### Distribution

South-western Amazonia.

#### Knodus breviceps

(Eigenmann 1908)

DAF0EBD6-A3E5-570D-902F-2E414020A097

##### Distribution

Eastern Peru.

##### Notes

Barriga (1994), Escobar-Camacho et al. (2015).

#### Knodus cf. caquetae

B1A702D7-4E01-59B4-A292-2C63E95DD7EE

##### Distribution

Amazon, Caquetá.

#### Knodus delta

Géry 1972

B8EEBC54-6004-5538-8BC6-DB0CFACA4B31

##### Distribution

Eastern Ecuador.

##### Notes

Barriga (2011).

#### Knodus gamma

Géry 1972

344CD549-F24D-5F11-961E-66D78E40CECE

##### Distribution

Eastern Ecuador.

##### Notes

Barriga (1994), Barriga (2011).

#### Knodus megalops

Myers 1929

CE5F57CA-84A2-5B96-834E-445465A61C02

##### Distribution

Amazonia.

#### Tyttocharax cf. cochui

6A764DEE-B4CB-5199-8EC9-1F10585D9663

##### Distribution

Amazonia.

#### Tyttocharax madeirae

Fowler 1913

174769E3-785E-5D98-B313-4F971BCFA644

##### Distribution

Amazon tributaries.

#### Xenurobrycon heterodon

Weitzman & Fink 1985

D85750A4-8AE5-5EAF-BE40-834DBF188921

##### Distribution

Upper Amazon.

##### Notes

Barriga (1994).

#### Characidae

EC9CD2F5-1C27-5C2B-A5C2-4919D2AF0B49

#### Acestrocephalus boehlkei

Menezes 1977

A9C47022-CE3E-5E25-89CB-6DA489DB2DEB

##### Distribution

Upper Amazon.

##### Notes

Barriga (1994), Galacatos et al. (2004).

#### Acestrocephalus sardina

(Fowler 1913)

FB49637A-F513-53BA-AEDB-A66D0B053475

##### Distribution

Western Amazon, Orinoco.

#### Aphyocharax pusillus

Günther 1868

00E93063-346B-55BE-B196-ACAB84E0D1A8

##### Distribution

South-western Amazonia.

##### Notes

Barriga (1994), Barriga (2011), Escobar-Camacho et al. (2015), Valdiviezo-Rivera et al. (2018).

#### Charax caudimaculatus

Lucena 1987

875C2F35-7CDA-5BB2-B723-94A87F5D8862

##### Distribution

South-western Amazonia.

##### Notes

Galacatos et al. (2004), Escobar-Camacho et al. (2015).

#### Charax cf. michaeli

0EB21D2C-9A1C-59C1-B967-7164B430CD88

##### Distribution

South-western Amazonia.

#### Charax tectifer

(Cope 1870)

6557E4A2-4C02-564E-BC99-A7F2E19780F3

##### Distribution

Upper Amazon.

##### Notes

Jácome-Negrete et al. (2018).

#### Cynopotamus amazonum

(Günther 1868)

289F6540-B760-5FE0-B3ED-5BC3326EA5DE

##### Distribution

Amazonia.

##### Notes

Barriga (1994), Galacatos et al. (2004), Escobar-Camacho et al. (2015).

#### Galeocharax gulo

(Cope 1870)

90F64ECC-8DB5-5A50-90B3-11773BF2D83C

##### Distribution

Widespread Amazonia.

#### Heterocharax macrolepis

Eigenmann 1912

BC09937A-E0D6-5174-BE7C-78DC46E23B37

##### Distribution

Amazon, plus northern drainages.

##### Notes

Barriga (1994), Barriga (2011).

#### Leptagoniates steindachneri

Boulenger 1887

D39615B7-C619-50BA-85A6-2AE4ED95AB42

##### Distribution

Amazonia.

##### Notes

Barriga (1994).

#### Odontostilbe ecuadorensis

Bührnheim & Malabarba 2006

8FAF17F9-D1FD-572C-81EE-6C5CF229A4FC

##### Distribution

Eastern Ecuador, Napo River Basin in Peru.

#### Odontostilbe fugitiva

Cope 1870

5A34F6FB-E0F7-5FB5-9A82-E65B408D425E

##### Distribution

Western Amazonia.

#### Odontostilbe roloffi

Géry 1972

7C21CA84-9150-5252-9F26-372A8FE25E82

##### Distribution

Napo Basin in Ecuador.

##### Notes

Barriga (1994).

#### Paragoniates alburnus

Steindachner 1876

B60B29D9-EF26-582F-B6B5-B28CD3064F4D

##### Distribution

Middle and upper Amazonia.

##### Notes

Barriga (1994).

#### Phenacogaster pectinata

(Cope 1870)

19C4FF1F-0DD9-5EFE-B9E4-0473E2F1D91B

##### Distribution

Middle and upper Amazonia.

##### Notes

Barriga (1994), Escobar-Camacho et al. (2015).

#### Prionobrama filigera

(Cope 1870)

4246CB67-8407-52F6-97FE-0E8E7C6AF802

##### Distribution

Amazonia.

##### Notes

Barriga (1994), Escobar-Camacho et al. (2015).

#### Roeboides dispar

Lucena 2001

7F796A83-68E6-56A6-A999-0DE831362B1C

##### Distribution

South-western Amazonia.

#### Roeboides myersii

Gill 1870

4B5759E0-6B84-5931-B4A5-46C8969924DE

##### Distribution

Amazonia, Orinoco.

##### Notes

Barriga (1994), Galacatos et al. (2004), Valdiviezo-Rivera et al. (2018).

#### Tetragonopterus argenteus

Cuvier 1816

88D86662-2394-544C-AF39-D789E403AF95

##### Distribution

Amazon, Orinoco, Paraguay.

##### Notes

Barriga (1994), Galacatos et al. (2004), Jácome-Negrete (2013), Escobar-Camacho et al. (2015), Jácome-Negrete et al. (2018), Valdiviezo-Rivera et al. (2018).

#### Tetragonopterus chalceus

Spix & Agassiz 1829

A4C935B0-C89A-5FA6-AB7E-E01C00F35CCE

##### Distribution

Amazon, plus northern drainages.

##### Notes

Galacatos et al. (2004).

#### Acestrorhamphidae

3ED41250-27D4-5CB5-8C26-0A7968000152

#### Astyanax abramis

(Jenyns 1842)

C8AE8C4F-34DF-5BBF-B327-6B6E2E843433

##### Distribution

Upper Amazon.

##### Notes

Barriga (1994), Valdiviezo-Rivera et al. (2018).

#### Astyanax bimaculatus

(Linnaeus 1758)

88E99A83-AAC1-5FB1-B63D-38CA174FC067

##### Distribution

Widespread Amazonia.

##### Notes

Barriga (1994), Valdiviezo-Rivera et al. (2018).

#### Astyanax bourgeti

Eigenmann 1908

340FD23B-B6E5-53A4-AFA1-4BB20D9F2571

##### Distribution

Upper Amazon.

#### Astyanax maximus

(Steindachner 1876)

FEF46B59-F62E-5F31-BD5D-0A90464A028C

##### Distribution

Upper Amazon, Orinoco.

##### Notes

Barriga (1994), Escobar-Camacho et al. (2015).

#### Astyanax sp. 1

135A2A10-E946-571B-A88F-015EC4C9191C

##### Distribution

Eastern Ecuador, unnamed river tributary of the Dicaro and Yasuní Rivers.

#### Bario steindachneri

(Eigenmann 1893)

B78B94BD-C126-5D2A-95A1-00615CA82827

##### Distribution

Amazonia.

##### Notes

Barriga (1994).

#### Bario oligolepis

(Günther 1864)

E3CB2261-6837-5EB5-9823-976BEC4C3D50

##### Distribution

Amazon, plus northern drainages.

##### Notes

Escobar-Camacho et al. (2015), Valdiviezo-Rivera et al. (2018), Tobes et al. (2022).

#### Brachychalcinus nummus

Böhlke 1958

63A56D92-576C-5472-B7F8-38EFB43357F7

##### Distribution

Upper Amazon.

##### Notes

Barriga (1994), Escobar-Camacho et al. (2015).

#### Bryconella pallidifrons

(Fowler 1946)

FE40DEF1-09C7-568A-9481-668B34E0DF40

##### Distribution

Amazonia.

##### Notes

Galacatos et al. (2004).

#### Ctenobrycon hauxwellianus

(Cope 1870)

5E8B16BA-E602-5736-9195-EB5AF9233967

##### Distribution

Amazonia.

##### Notes

Galacatos et al. (2004), Escobar-Camacho et al. (2015), Valdiviezo-Rivera et al. (2018), Jácome-Negrete et al. (2018).

#### Gymnocorymbus thayeri

Eigenmann 1908

D051787F-19F5-5CF9-AD64-793108BFB9D6

##### Distribution

Western Amazonia, plus northern drainages.

##### Notes

Barriga (1994), Escobar-Camacho et al. (2015).

#### Hemigrammus bellottii

(Steindachner 1882)

F80EF1C9-5562-5122-97D0-AC22814E2F7D

##### Distribution

Western Amazonia.

#### Hemigrammus collettii

(Steindachner 1882)

00B6C8AB-984B-534D-A04E-5650197DC74B

##### Distribution

Amazon, plus northern drainages.

#### Hemigrammus coeruleus

Durbin 1908

F31BA92C-B453-5FB7-AA57-B9E1C1AF457F

##### Distribution

Western Amazonia.

#### Hemigrammus cupreus

Durbin 1918

07881801-4ED9-5714-B86A-F9C81F2CCC6A

##### Distribution

Western Amazonia.

##### Notes

Galacatos et al. (2004).

#### Hemigrammus hyanuary

Durbin 1918

F5A1AF46-B52F-5975-BAC6-EDCE3FDBCC2A

##### Distribution

Amazonia.

#### Hemigrammus levis

Durbin 1908

6416D1EE-E52A-5EEE-968E-594978B9A760

##### Distribution

Amazonia.

#### Hemigrammus luelingi

(Géry 1964)

1A0E5B6F-8201-5550-8E17-58AA4C59505B

##### Distribution

Eastern Ecuador, eastern Peru.

##### Notes

Galacatos et al. (2004).

#### Hemigrammus lunatus

Durbin 1918

9DFB32DC-A55E-59DA-B375-522FAA630BB8

##### Distribution

Amazon, Orinoco, Paraguay.

##### Notes

Barriga (1994), Galacatos et al. (2004).

#### Hemigrammus cf. marginatus

57CA46E8-D7B0-57E7-81FD-748C4DB098B3

##### Distribution

Amazonia, Orinoco.

##### Notes

Barriga (2012).

#### Hemigrammus megaceps

Fowler 1945

48826277-50E1-5700-BB1A-AD3C54F9DD44

##### Distribution

Eastern Ecuador, eastern Peru.

#### Hemigrammus pretoensis

Géry 1965

6388FFE5-CED0-518E-A30D-C36067BBAAC6

##### Distribution

Upper Amazon.

##### Notes

Barriga (2012).

#### Hemigrammus unilineatus

(Gill 1858)

9ECC72F2-D915-5CEA-9005-BD7B5E0B8801

##### Distribution

Amazon, plus northern drainages.

##### Notes

Galacatos et al. (2004), Valdiviezo-Rivera et al. (2021).

#### Holopristis ocellifera

(Steindachner 1882)

819504F4-02FB-580D-BFE6-2CD0DFD6205A

##### Distribution

Amazon, plus northern drainages.

##### Notes

Galacatos et al. (2004).

#### Holopristis pulchra

(Ladiges 1938)

6F6C6080-E721-54FC-9FA2-FD3F342EA011

##### Distribution

Upper Amazon.

##### Notes

Barriga (2012).

#### Hyphessobrycon agulha

Fowler 1913

5B529A27-C916-5A33-9C3B-534FE3201042

##### Distribution

Western, south-western Amazonia.

#### Hyphessobrycon gracilior

Géry 1964

244EB21F-CF8C-5438-AECC-7C28C56B0913

##### Distribution

Upper Amazon.

#### Hyphessobrycon loretoensis

Ladiges 1938

0E71C103-9BD1-50C7-B5E9-132212C3BD3E

##### Distribution

Upper Amazon.

#### Hyphessobrycon tenuis

Géry 1964

441D8B0C-FBBF-54F7-B210-7A9871608748

##### Distribution

Upper Amazon.

#### Hyphessobrycon sp. 1

4253BF6D-2314-5877-8E6B-B5D7D40E91B2

##### Distribution

Eastern Ecuador, ravine Cotoyacu, confluence with Yasuni River.

##### Notes

Galacatos et al. (2004).

#### Hyphessobrycon sp. 2

92C90DCD-30CF-58A0-A883-524F4AFC87D1

##### Distribution

Eastern Ecuador, Yasuní River at the confluence with Jatuncocha Lagoon.

#### Jupiaba anteroides

(Géry 1965)

87361698-3629-5A0A-902B-6FFA74BF7504

##### Distribution

Upper Amazon.

#### Jupiaba asymmetrica

(Eigenmann 1908)

36F6AED3-0D74-569D-A759-C9F7BBB1FBB5

##### Distribution

Upper Amazon.

##### Notes

Barriga (1994).

#### Jupiaba zonata

(Eigenmann 1908)

1B9FDAF5-93DC-5CEB-8E88-59150F0903F3

##### Distribution

Upper Amazon, Rio Negro.

#### Megalamphodus copelandi

(Durbin 1908)

FB0B120A-5B65-5131-9EDF-0F18FBDFC776

##### Distribution

Upper Amazon.

##### Notes

Barriga (1994), Galacatos et al. (2004), Escobar-Camacho et al. (2015).

#### Megalamphodus bentosi

(Durbin 1908)

4BCA369C-D1B0-5E03-8644-27367247748B

##### Distribution

Amazonia.

##### Notes

Galacatos et al. (2004).

#### Moenkhausia chrysargyrea

(Günther 1864)

03D7BDDB-523D-52DE-9A03-12EF23530223

##### Distribution

Amazon, plus northern drainages.

##### Notes

Barriga (1994).

#### Moenkhausia comma

Eigenmann 1908

099B35E7-FF0A-538C-96DE-AF5F222C6ADE

##### Distribution

Amazonia.

##### Notes

Barriga (1994), Escobar-Camacho et al. (2015).

#### Moenkhausia aff. copei

FCD6C9BF-DCF9-58D0-BEE3-5F8FBE621DC0

##### Distribution

Amazonia, Orinoco.

##### Notes

Escobar-Camacho et al. (2015).

#### Moenkhausia cotinho

Eigenmann 1908

C18C3CD1-1C9D-538C-96D5-0764058C0353

##### Distribution

Amazonia

##### Notes

Galacatos et al. (2004), Escobar-Camacho et al. (2015).

#### Moenkhausia cf. dichroura

E65C1181-6331-57F7-91DA-E4BA40CAF3FE

##### Distribution

Amazon, Orinoco, Paraguay.

##### Notes

Galacatos et al. (2004), Valdiviezo-Rivera et al. (2018), Tobes et al. (2022).

#### Moenkhausia grandisquamis

Müller & Troschel, 1845

890137DC-9E71-5A9C-8D48-07AB8AB2CE56

##### Distribution

Amazon, plus northern drainages.

##### Notes

Barriga (1994), Escobar-Camacho et al. (2015).

#### Moenkhausia latissima

Eigenmann 1908

A6818C79-E031-538A-A640-EC14183D7640

##### Distribution

Upper Amazon.

#### Moenkhausia lepidura

(Kner 1858)

F09DD553-A2C2-519E-AA2D-0DF394335C68

##### Distribution

Amazon, plus northern drainages.

##### Notes

Tobes et al. (2022).

#### Moenkhausia naponis

Böhlke 1958

55BA6490-FFA2-58C4-9D7D-35404862BF14

##### Distribution

Eastern Ecuador.

##### Notes

Barriga (1994).

#### Moenkhausia ovalis

(Günther 1868)

F2F6DCA1-AA29-58D7-B9CD-8AA0FBB3F79F

##### Distribution

Upper Amazon.

#### Moenkhausia simulata

(Eigenmann 1924)

2F63DFD8-5746-5524-9865-5E25FD8345BA

##### Distribution

Upper Amazon.

#### Poptella compressa

(Günther 1864)

8ADA93A4-AD27-5FE6-A4A6-9168B438B572

##### Distribution

Amazon, plus northern drainages.

##### Notes

Barriga (1994), Galacatos et al. (2004).

#### Psalidodon henseli

(de Melo & Buckup 2006)

95AFE29E-C384-536E-8966-8BE2970C4D54

##### Notes

Valdiviezo-Rivera et al. (2018), Tobes et al. (2022).

#### Stethaprion erythrops

Cope 1870

3454FBF1-A852-56F3-A107-48E36700B5E9

##### Distribution

Upper Amazon.

##### Notes

Barriga (1994), Galacatos et al. (2004).

#### Thayeria obliqua

Eigenmann 1908

A195559A-2BC3-5549-BE6D-1F556E346BE0

##### Distribution

Western and southern Amazonia.

##### Notes

Barriga (1994).

#### Tyttobrycon hamatus

Géry 1973

59A51EFD-D315-590B-94B8-AEB246509268

##### Distribution

Amazonia.

#### Gymnotiformes

040C4A91-2E6A-53D2-8AE2-9B6B62711963

#### Apteronotidae

B7708595-A42B-58B8-A3A2-D1E0BBFB58FE

#### Adontosternarchus balaenops

(Cope 1878)

6012CA95-2E9D-541B-AB60-9E1078ED3D73

##### Distribution

Upper Amazon.

#### Adontosternarchus clarkae

Mago-Leccia, Lundberg & Baskin 1985

448E96FC-3367-58B8-B6AD-FD0CAAEE15AF

##### Distribution

Amazonia, Orinoco.

##### Notes

Barriga (1994).

#### Apteronotus albifrons

(Linnaeus 1766)

DE3056C8-1DF9-5771-AC82-82730C0505C5

##### Distribution

Amazon, plus northern drainages.

##### Notes

Barriga (1994), Barriga (2011), Guarderas and Jácome-Negrete (2013).

#### Apteronotus apurensis

Fernández-Yépez 1968

5E3DA4B4-5710-5D47-9789-23A4123181B7

##### Distribution

Orinoco drainage.

#### Apteronotus bonapartii

(Castelnau 1855)

2B625420-D7E3-53EB-96DE-8C6D4BDAA3B7

##### Distribution

Amazon, larger rivers.

#### Porotergus cf. duende

26482C58-8F13-5CF9-AC72-87E66B633181

##### Distribution

Western Amazonia, larger rivers.

#### Sternarchorhynchus curvirostris

(Boulenger 1887)

0D18DAC5-D749-5746-BD34-06CAB01AC91C

##### Distribution

Eastern Ecuador, north-eastern Peru.

##### Notes

Barriga (1994), Guarderas and Jácome-Negrete (2013).

#### Sternarchorhynchus cf. oxyrhynchus

B39700DE-A51B-55FA-AC21-030CDB9D6F7B

##### Distribution

Amazon, plus northern drainages.

#### Sternopygidae

F5D42BF1-3067-5896-8557-DFCBD7B370AA

#### Distocyclus conirostris

(Eigenmann & Allen 1942)

7AE330C3-60E0-5FF8-B968-936F0DB45CE0

##### Distribution

Amazonia, Orinoco.

#### Eigenmannia cf. limbata

498E12E1-71A8-5690-965B-536C3FE39776

##### Distribution

Amazon, plus northern drainages.

##### Notes

Galacatos et al. (2004).

#### Eigenmannia cf. virescens

FB06BCEC-F309-57DF-9B05-E98D04BE8E94

##### Distribution

Widespread Amazonia, La Plata.

##### Notes

Barriga (1994), Barriga (2011), Guarderas and Jácome-Negrete (2013), Jácome-Negrete (2013), Jácome-Negrete et al. (2018).

#### Rhabdolichops eastwardi

Lundberg & Mago-Leccia 1986

8F7C491F-6D1D-568C-B72B-D356E2A4F71F

##### Distribution

Amazonia, Orinoco.

#### Sternopygus macrurus

(Bloch & Schneider 1801)

62260F9C-D8F7-5546-892E-AA9C38860A9D

##### Distribution

Widespread Amazonia.

##### Notes

Barriga (1994), Galacatos et al. (2004), Barriga (2011), Guarderas and Jácome-Negrete (2013).

#### Gymnotidae

0AA9D511-E2F3-5D76-A1B3-DFD0C170A455

#### Electrophorus varii

de Santana, Wosiacki, Crampton, Sabaj, Dillman, Mendes-Júnior & Castro e C. 2019

120DDDAB-4A48-5782-9231-DB8028F20FDA

##### Distribution

Western and central Amazonia.

##### Notes

Barriga (1994), Galacatos et al. (2004), Barriga (2011), Guarderas and Jácome-Negrete (2013), Jácome-Negrete (2013), Jácome-Negrete et al. (2018).

#### Gymnotus carapo

Linnaeus, 1758

1392E5E6-2B5B-56FA-90D0-57220F05F137

##### Distribution

Mexico to Paraguay.

##### Notes

Barriga (1994), Galacatos et al. (2004), Barriga (2011), Guarderas and Jácome-Negrete (2013).

#### Gymnotus coatesi

LaMonte 1935

3FB2911D-CCD0-54F7-80D1-5EEA93CAB357

##### Distribution

Widespread Amazonia, western piedmont tributaries to main channel.

##### Notes

Barriga (1994).

#### Gymnotus sp. 1

E93E82A7-21A9-52FB-8895-85236BB22A73

##### Distribution

Eastern Ecuador, Tambococha ravine.

#### Hypopomidae

615F5061-1C92-5375-B51A-DB18E678DF9A

#### Brachyhypopomus beebei

(Schultz 1944)

04DE6B0A-A857-57CB-8F26-032DB58B84E0

##### Distribution

Western Amazonia.

##### Notes

Galacatos et al. (1996).

#### Brachyhypopomus brevirostris

(Steindachner 1868)

A6AEEFC4-770B-550D-A106-4184F1AD2781

##### Distribution

Western Amazonia, Orinoco, La Plata.

#### Brachyhypopomus bennetti

Sullivan, Zuanon & Cox Fernandes 2013

340446BE-A003-5799-9C50-498273BE8D91

##### Distribution

Amazonia.

#### Brachyhypopomus pinnicaudatus

(Hopkins, Comfort, Bastian & Bass 1990)

A1274BFD-D6F5-5ED3-AB3C-A3EB944E3889

##### Distribution

Amazon, plus northern drainages, La Plata.

#### Brachyhypopomus regani

Crampton, de Santana, Waddell & Lovejoy 2017

2EB5927E-AF92-5813-929B-BCE4A34A5E67

##### Distribution

Amazon, plus northern drainages.

#### Brachyhypopomus walteri

Sullivan, Zuanon & Cox Fernandes 2013

184D8E06-B757-5CF1-AC4C-6C4DBC39C6A1

##### Distribution

Amazonia.

#### Rhamphichthyidae

33DB6526-C8FE-5535-A3EF-68DE35864FC1

#### Gymnorhamphichthys hypostomus

Ellis 1912

EABA97B2-9E60-5137-9DE3-41C3859D582A

##### Distribution

Western Amazonia, Orinoco.

##### Notes

Barriga (1994), Guarderas and Jácome-Negrete (2013).

#### Gymnorhamphichthys sp. 1

6C20CE61-40D4-553E-8C7D-EF964A4AAD00

##### Distribution

Eastern Ecuador, Cononaco River, close to the community Bameno.

#### Hypopygus lepturus

Hoedeman 1962

A83C3939-9562-59D0-A173-5E5A31CA548E

##### Distribution

Amazonia, Orinoco, Guianas.

##### Notes

Barriga (1994), Barriga (2011), Guarderas and Jácome-Negrete (2013).

#### Steatogenys elegans

(Steindachner 1880)

FE71B5E7-5FD5-5F1D-9021-016650AD637B

##### Distribution

Amazon, plus northern drainages.

##### Notes

Barriga (1994), Galacatos et al. (2004).

#### Siluriformes

0DCC9536-CDD1-5C4E-8DD9-966B02B41803

#### Trichomycteridae

F18B5BDF-EE3B-5CC2-A8EA-03674CA140B0

#### Henonemus punctatus

(Boulenger 1887)

90310CAC-FD72-5FE5-9B3F-9CDA8F2EB8F7

##### Distribution

Amazonia.

##### Notes

Barriga (1994), Barriga (2011).

#### Ochmacanthus reinhardtii

(Steindachner 1882)

8C2DE36D-2EE5-5192-B841-BD3F8A1256AF

##### Distribution

Amazon, plus northern drainages.

#### Ochmacanthus sp. 1

80294CC9-DEA1-559E-82C8-A1A980B656A1

##### Distribution

Eastern Ecuador, Yasuni River, confluence with Jatuncocha River.

#### Paracanthopoma parva

Giltay 1935

A0B7C9E1-EB7D-5A83-B795-16B79A450CAB

##### Distribution

Amazonia.

##### Notes

Barriga (1994).

#### Paravandellia sp. 1

AD5954A9-280A-5605-9097-35196E0EE690

##### Distribution

Eastern Ecuador, Jatuncocha lagoon.

#### Plectrochilus wieneri

(Pellegrin 1909)

BF0FB8CF-8B16-5C7B-8E9A-468936F2BE75

##### Distribution

Napo Basin in Ecuador.

##### Notes

Barriga (1994).

#### Pseudostegophilus nemurus

(Günther 1869)

9071E980-66D6-51CB-B558-16B49D3F7F96

##### Distribution

Amazonia.

##### Notes

Barriga (1994), Barriga (2011), Guarderas and Jácome-Negrete (2013).

#### Schultzichthys bondi

(Myers 1942)

09197BFB-D1C2-51CC-9A81-BB0EA4B5B05C

##### Distribution

Amazonia, Orinoco.

##### Notes

Barriga (1994), Barriga (2011).

#### Tridens melanops

Eigenmann & Eigenmann 1889

59A1EACD-5506-552C-BBE0-BA61DF2E20A0

##### Distribution

Western and central Amazonia.

#### Tridentopsis pearsoni

Myers 1925

42051215-140D-5D55-837F-E88A48967A24

##### Distribution

Western, south-western Amazonia.

##### Notes

Galacatos et al. (2004).

#### Vandellia cirrhosa

Valenciennes 1846

96FB7436-721C-59DB-ACFD-08B9D7DA55F4

##### Distribution

Widespread Amazonia.

##### Notes

Barriga (2011).

#### Callichthyidae

542466DB-DB1A-56D2-B10D-879F4F9706FF

#### Brochis ambiacus

(Cope 1872)

F879F234-B5F0-5A6D-996B-FD1B900DA894

##### Distribution

Upper Amazon

##### Notes

Barriga (1994), Guarderas and Jácome-Negrete (2013).

#### Brochis arcuatus

(Elwin 1938)

3F2A35AE-4B87-5347-92AB-93005D7D2C6C

##### Distribution

South America: Upper Amazon River Basin.

#### Brochis leopardus

(Myers 1933)

9031E8F1-2023-50E1-8538-15F06563D6F5

##### Distribution

Western Amazonia.

#### Brochis splendens

(Castelnau 1855)

31B3FB7E-6683-5FE5-B7AC-92B71862B1E7

##### Distribution

Amazonia.

#### Callichthys callichthys

(Linnaeus 1758)

F161612C-8107-5051-8C54-B0FBEB1C1BD3

##### Distribution

Widespread Amazonia.

##### Notes

Barriga (1994), Galacatos et al. (2004), Barriga (2011), Guarderas and Jácome-Negrete (2013).

#### Corydoras acutus

Cope 1872

4A8F43E6-965D-5FA2-A711-C16FA01265FC

##### Distribution

Eastern Ecuador, north-eastern Peru, southern Brazil.

##### Notes

Barriga (1994), Barriga (2011), Guarderas and Jácome-Negrete (2013).

#### Dianema longibarbis

Cope 1872

9D62929A-A841-5F28-8363-E1A5A78C2931

##### Distribution

Amazonia.

##### Notes

Barriga (1994), Galacatos et al. (2004).

#### Gastrodermus elegans

(Steindachner 1876)

DA011682-7186-592F-8E9A-FF833D419872

##### Distribution

Upper Amazon.

##### Notes

Barriga (1994).

#### Gastrodermus napoensis

(Nijssen & Isbrücker 1986)

A41BD2E1-9EAD-5736-B6B1-A11D82A2EC9D

##### Distribution

Eastern Ecuador, north-eastern Peru.

#### Hoplisoma armatum

(Günther 1868)

D972DE64-5D75-5A64-9843-4834D5A90B0D

##### Distribution

Upper Amazon.

#### Hoplisoma leucomelas

(Eigenmann & Allen 1942)

BDD51001-5CB9-579A-BBFC-5A256B664282

##### Distribution

Upper Amazon.

#### Hoplisoma cf. loretoense

03A1705C-B99A-54A7-A16F-E2BF62C85D98

##### Distribution

Upper Amazon.

#### Hoplisoma trilineatum

(Cope 1872)

2BF615E9-4A96-55BF-A7CA-D3574985CF0C

##### Distribution

Amazon, plus northern drainages.

#### Hoplisoma cf. melini

E16FB245-256A-50E0-AE77-B94678F41DCF

##### Distribution

Eastern Ecuador, south-eastern Colombia, Rio Negro.

#### Hoplosternum littorale

(Hancock 1828)

0B743292-6715-5F75-BAFA-EE68F524AD91

##### Distribution

Widespread Amazonia.

##### Notes

Galacatos et al. (2004), Barriga (2011).

#### Megalechis thoracata

(Valenciennes 1840)

E5D4FBA8-3A3F-5514-A62E-6E2C95B3011C

##### Distribution

Amazon, plus northern drainages.

##### Notes

Galacatos et al. (2004).

#### Osteogaster aenea

(Gill 1858)

519FF6EC-E5E5-5BC2-8C3A-0963E2CD7A5C

##### Distribution

Widespread Amazonia, La Plata.

##### Notes

Guarderas and Jácome-Negrete (2013).

#### Osteogaster eques

(Steindachner 1876)

A3FE7EA3-67F2-5581-A66B-4F52B92F2CA7

##### Distribution

Amazonia.

#### Osteogaster rabauti

(LaMonte 1941)

867C05E1-9FB1-5F81-AC65-046B581D27C6

##### Distribution

Upper Amazon.

#### Loricariidae

53E4681F-9404-553E-8695-1F562686C5E0

#### Ancistrus alga

(Cope 1872)

6817ADF9-1B86-5D62-B485-1FF13F8BC0A1

##### Distribution

Amazon, plus northern drainages, Paraguay.

##### Notes

Galacatos et al. (2004).

#### Ancistrus malacops

(Cope 1872)

5EA4172E-0E18-58F6-8E19-F0DE56AB019C

##### Notes

Tobes et al. (2022).

#### Aphanotorulus unicolor

(Steindachner 1908)

2322957D-E89A-5E23-B430-F9001839F634

##### Distribution

Upper Amazon.

##### Notes

Barriga (2011), Guarderas and Jácome-Negrete (2013), Jácome-Negrete (2013), Jácome-Negrete et al. (2018).

#### Chaetostoma branickii

Steindachner 1881

3DC20562-DF89-52D4-AF45-659752BAB0A6

##### Distribution

Marañon River basin, Ecuador and Peru.

#### Chaetostoma microps

Günther 1864

9448E7CB-EC90-561A-AF0C-23BBE173C674

##### Notes

Tobes et al. (2022).

#### Farlowella knerii

(Steindachner 1882)

C74C8A78-3468-585D-845C-59B93C0FF9B4

##### Distribution

Upper Amazon, Napo, Pastaza.

##### Notes

Guarderas and Jácome-Negrete (2013).

#### Farlowella nattereri

Steindachner 1910

D39F3057-BFC3-5547-9909-D7B09EE90941

##### Distribution

Upper Amazon, Essequibo.

##### Notes

Barriga (2011).

#### Farlowella odontotumulus

Retzer & Page 1997

CDBF4E97-AE4E-5506-85C6-C6D79264ACAC

##### Distribution

Eastern Ecuador, Venezuela.

#### Farlowella oxyrryncha

(Kner 1853)

C168570D-6F46-5841-80B7-03D369693F62

##### Distribution

Amazonia, Orinoco.

#### Hypoptopoma thoracatum

Günther 1868

BAD07897-9428-571B-8758-309B393DE1F1

##### Distribution

Amazonia.

##### Notes

Barriga (2011).

#### Hypoptopoma gulare

Cope 1878

CEB52688-E953-5FC6-B5EA-D5E3FE85FE90

##### Distribution

Amazonia.

#### Hypoptopoma sp. 1

B95F6531-62C8-5294-8A59-A1DB5F7CDFE5

##### Distribution

Eastern Ecuador, Yasuní River, confluence with Jatuncocha River.

##### Notes

Jácome-Negrete et al. (2018).

#### Hypostomus ericius

Armbruster 2003

0BA333BF-1E99-581E-911C-46390490A2EF

##### Distribution

Upper Amazon.

#### Hypostomus hemicochliodon

Armbruster 2003

31086183-15A1-575D-A5A6-CDFF11B53573

##### Distribution

Upper Amazon, upper Orinoco, upper Rio Negro.

##### Notes

Tobes et al. (2022).

#### Hypostomus oculeus

(Fowler 1943)

E96898C0-A311-5336-AFF7-45F9B7DE32DA

##### Distribution

North-western Amazon.

##### Notes

Barriga (2011).

#### Hypostomus pyrineusi

(Miranda Ribeiro 1920)

32A13E76-B297-5668-9A04-251354735A45

##### Distribution

South-western Amazon.

#### Hypostomus sp. 1

CBC33A18-E148-5A9B-A524-E266E263A8A9

##### Distribution

Eastern Ecuador, pond Sachacocha, affluent of Jatuncocha River.

##### Notes

Galacatos et al. (2004).

#### Lamontichthys filamentosus

(LaMonte 1935)

A5FE89DA-BDCB-5962-8802-844C43AA2A11

##### Distribution

Western Amazonia.

##### Notes

Barriga (1994), Galacatos et al. (2004), Barriga (2011).

#### Lasiancistrus cf. heteracanthus

254A12B2-F088-5E0B-8548-DB17688362A4

##### Distribution

Rio Napo, Ecuador and Peru.

#### Lasiancistrus schomburgkii

(Günther 1864)

5F4D00AD-8C5D-50B3-90A4-8CAFA498076E

##### Distribution

Amazon, plus northern drainages.

##### Notes

Tobes et al. (2022).

#### Limatulichthys griseus

(Eigenmann 1909)

3C33847B-8187-52D1-A4D1-B89716756BAA

##### Distribution

Amazon, plus northern drainages.

##### Notes

Barriga (1994), Barriga (2011).

#### Loricaria cataphracta

Linnaeus 1758

933D23F5-85B5-560C-B33A-92B602E9F531

##### Distribution

Amazon, plus northern drainages

#### Loricaria clavipinna

Fowler 1940

A1F63BF8-7773-57EA-9A52-B4196CDCEA62

##### Distribution

Amazonia.

##### Notes

Guarderas and Jácome-Negrete (2013).

#### Loricaria simillima

Regan 1904

B7D6FC4B-D9E2-5334-BBCF-B888299E90A5

##### Distribution

Amazonia, Orinoco, La Plata.

##### Notes

Barriga (1994), Galacatos et al. (2004), Barriga (2011), Guarderas and Jácome-Negrete (2013).

#### Loricariichthys cashibo

(Eigenmann & Allen 1942)

BB88EA24-9949-52EB-BEFC-DF24DFF9546B

##### Distribution

Upper Amazon.

#### Loricariichthys hauxwelli

Fowler 1915

C0A05023-A5DF-5069-924C-522967106034

##### Distribution

South-eastern Ecuador, north-eastern Peru.

#### Nannoptopoma spectabile

(Eigenmann 1914)

3196A9AA-45D0-554C-B492-F6F82B4E988C

##### Distribution

Amazonia, Orinoco.

#### Nannoptopoma sternoptychum

Schaefer 1996

6E8257F0-489B-5DE9-88CC-71F9D85BCEE0

##### Distribution

Upper Amazon.

#### Otocinclus batmani

Lehmann A. 2006

068DE2F2-07FD-5A49-A685-B102AD059B94

##### Distribution

Disjunct patches in western Amazon.

#### Otocinclus huaorani

Schaefer 1997

4E1C8B5F-B33B-53DC-B9A9-8679223D626D

##### Distribution

Upper Amazon, Orinoco.

##### Notes

Galacatos et al. (2004), Barriga (2011).

#### Otocinclus macrospilus

Eigenmann & Allen 1942

FC356970-4F5B-562D-8DFF-045D638053EF

##### Distribution

Amazonia.

##### Notes

Galacatos et al. (2004) .

#### Otocinclus cf. hoppei

AC40EE6B-D01B-55BC-A69F-C4C725702BD9

##### Distribution

Easternn Ecuador, Jatuncocha Lake.

#### Panaqolus albomaculatus

(Kanazawa 1958)

CBA2403D-B896-5911-9760-28DEA5A7C1E8

##### Distribution

Upper Napo, eastern Peru.

##### Notes

Barriga (1994), Barriga (2011).

#### Panaqolus dentex

(Günther 1868)

B17BA44C-0D3E-5C4C-8C95-C5683221BD1D

##### Distribution

Napo, eastern Ecuador, eastern Peru.

##### Notes

Provenzano-Rizzi et al. (2024).

#### Panaqolus nocturnus

(Schaefer & Stewart 1993)

8E1E0C2B-7F14-5FA8-A56E-A4DF0B2C21BA

##### Distribution

Napo drainage, Ecuador.

##### Notes

Barriga (2011).

#### Panaqolus orcesi

Provenzano, Barriga-Salazar & Stewart 2024

2F342E19-CFAE-55A7-8B7E-AD3DB52A27F4

##### Distribution

Ecuadorian Amazon.

##### Notes

Provenzano-Rizzi et al. (2024).

#### Panaque nigrolineatus

(Peters 1877)

FAE4AA36-010C-5D5B-A997-3572A2E70528

##### Distribution

Amazonia, Orinoco.

##### Notes

Barriga (1994), Barriga (2011).

#### Panaque schaeferi

Lujan, Hidalgo & Stewart 2010

C175DC9B-AA7C-5298-8198-8C4DC4C97EAE

##### Distribution

Upper Amazon.

##### Notes

Lujan et al. (2010).

#### Panaque titan

Lujan, Hidalgo & Stewart 2010

D1EC0774-A271-5F2E-B4B5-44C1F381D9E1

##### Distribution

Napo Basin, Ecuador.

##### Notes

Lujan et al. (2010).

#### Peckoltia bachi

(Boulenger 1898)

E52A7CF6-C48C-5684-950A-3FDCEC9074F1

##### Distribution

Upper Amazon.

#### Peckoltia furcata

(Fowler 1940)

CEF08B02-CA5A-5983-AEC1-86F1A8C1E9B6

##### Distribution

Upper Amazon.

##### Notes

Galacatos et al. (2004), Barriga (2011), Guarderas and Jácome-Negrete (2013).

#### Planiloricaria cryptodon

(Isbrücker 1971)

7FDFEA07-50B8-5FD1-AAF5-B6B0508D9275

##### Distribution

Upper Amazonia.

#### Pseudohemiodon apithanos

Isbrücker & Nijssen 1978

E5AAABD0-E14B-5699-83BF-B02B937D8210

##### Distribution

Western Amazonia.

##### Notes

Barriga (1994).

#### Pseudohemiodon lamina

(Günther 1868)

A5AC4345-AC93-5295-8E49-04ED3FEAA0B9

##### Distribution

Upper Amazon.

##### Notes

Provenzano-Rizzi and Barriga-Salazar (2020).

#### Pterygoplichthys gibbiceps

(Kner 1854)

196FB8F7-6DE2-54A3-A10C-812C91A546E3

##### Distribution

Middle and upper Amazon, Orinoco.

#### Pterygoplichthys pardalis

(Castelnau 1855)

6887964A-8336-5FF6-BD3C-1F1F25C0CB26

##### Distribution

Widespread Amazonia.

##### Notes

WCS (2007), Valdiviezo-Rivera et al. (2018).

#### Pterygoplichthys punctatus

(Kner 1854)

05FB3598-2DA5-5BCC-9275-3E1059283383

##### Distribution

Western Amazonia.

#### Pterygoplichthys weberi

Armbruster & Page 2006

FE27A0D6-C1E3-5091-81AD-54E7CB4C4B2A

##### Distribution

Upper Amazon of Colombia, Ecuador, Peru.

##### Notes

Valdiviezo-Rivera et al. (2018).

#### Rhadinoloricaria condei

(Isbrücker & Nijssen 1986)

E55B0411-F3D7-582D-8019-DF09B9E63CAB

##### Distribution

Napo Basin, Ecuador.

#### Rhadinoloricaria macromystax

(Günther 1869)

1988EAA9-D5C3-57CE-A1B8-065E6F51D70E

##### Distribution

Eastern Peru and Ecuador.

##### Notes

Barriga (1994).

#### Rineloricaria lanceolata

(Günther 1868)

3FFD5644-5AC7-5839-AF44-D79D73B223F6

##### Distribution

Upper Amazon.

##### Notes

Barriga (1994).

#### Rineloricaria morrowi

Fowler 1940

D2628268-4D86-5CFF-A386-FC84A48E7F7E

##### Distribution

Upper Amazon of Ecuador, Peru.

#### Spatuloricaria puganensis

(Pearson 1937)

DCF04F8C-88A1-5FDE-B823-4294915F4863

##### Distribution

Upper Amazon of Ecuador, Peru.

#### Sturisoma guentheri

(Regan 1904)

C07FD6FB-ED91-538F-8471-A76185F8B0EF

##### Distribution

Upper Amazon.

##### Notes

Galacatos et al. (2004).

#### Sturisoma nigrirostrum

Fowler 1940

EA253CC0-16CD-5EB6-A368-61E3D49A7934

##### Distribution

Upper Amazon of Ecuador, Peru.

##### Notes

Barriga (1994), Barriga (2011), Guarderas and Jácome-Negrete (2013).

#### Cetopsidae

0DC3A0CD-73A1-5502-AECB-A5832F99032B

#### Cetopsis candiru

Spix & Agassiz 1829

AF6086AC-E50F-587C-ADCD-81C964EEFB6F

##### Distribution

Amazonia.

##### Notes

Barriga (2011).

#### Cetopsis coecutiens

(Lichtenstein 1819)

4B4EAB36-9BA1-5646-80AD-9F3277AC5AFB

##### Distribution

Amazon, Orinoco.

##### Notes

Barriga (1994), Barriga (2011), Guarderas and Jácome-Negrete (2013).

#### Cetopsis oliveirai

(Lundberg & Rapp Py-Daniel 1994)

B0B7CEB7-02F6-5C4E-801D-F7C785A78BC9

##### Distribution

Middle and upper Amazon.

#### Cetopsis plumbea

Steindachner 1882

A070A78C-7F74-5DED-866D-69654B5E8862

##### Distribution

Upper Amazon.

#### Denticetopsis seducta

Vari, Ferraris & de Pinna 2005

0B392D69-3A12-5B15-963F-5A43833EF13D

##### Distribution

Amazon, Orinoco.

#### Helogenes marmoratus

Günther 1863

596315D8-B22B-57BA-8558-F03938451D27

##### Distribution

Amazon, plus northern drainages.

##### Notes

Guarderas and Jácome-Negrete (2013).

#### Aspredinidae

24FA65C7-4075-5B13-9C70-3D9424DAD7D8

#### Bunocephalus coracoideus

(Cope 1874)

436E297E-B9B1-5D0B-BDAF-10B08014248E

##### Distribution

Amazonia.

##### Notes

Guarderas and Jácome-Negrete (2013).

#### Bunocephalus knerii

Steindachner 1882

874C419B-562B-57C5-98C0-AC1FCAB39263

##### Distribution

Western Amazonia.

#### Bunocephalus verrucosus

(Walbaum 1792)

901B83D4-9BD8-5A54-B032-DD1130539771

##### Distribution

Amazon, plus northern drainages.

##### Notes

Galacatos et al. (2004).

#### Ernstichthys intonsus

Stewart 1985

FC7FBFE3-6563-5722-8EA1-8BA3FD7EF2A0

##### Distribution

Napo Basin, Ecuador.

##### Notes

Barriga (1994).

#### Micromyzon akamai

Friel & Lundberg 1996

18E33B4D-AEF7-53A3-B2F9-A7004BC0D2DC

##### Distribution

Amazon, Madeira, Napo, main channel and direct tributaries.

##### Notes

Chuctaya et al. (2021).

#### Pseudobunocephalus amazonicus

(Mees 1989)

8F2E5715-9473-500F-A2EB-1E0E85EC9344

##### Distribution

Upper and middle Amazon.

#### Pseudobunocephalus bifidus

(Eigenmann 1942)

2A18A8BB-6CF1-5A63-A2AE-4F0B2C15B751

##### Distribution

Amazonia.

##### Notes

Barriga (1994), Galacatos et al. (2004).

#### Pterobunocephalus depressus

(Haseman 1911)

2ADEBAC0-628E-59A4-9DC3-FF8FC7B58F93

##### Distribution

Amazon, Orinoco, Paraguay Basins.

#### Xyliphius lepturus

Orcés V. 1962

A7B9885F-E4B6-5F24-88B8-80526ACC4740

##### Distribution

Upper Amazon, Orinoco.

##### Notes

Barriga (1994).

#### Xyliphius melanopterus

Orcés V. 1962

6B25B5F8-4EE4-59F9-92E6-2125BCF0C068

##### Distribution

Amazon, Orinoco.

##### Notes

Barriga (1994).

#### Auchenipteridae

FC9E9B29-AADF-54D1-80EF-7889AD6377F7

#### Ageneiosus inermis

(Linnaeus 1766)

EA25B247-6876-5F27-A289-CD2485CEC3DB

##### Distribution

Most of S.A. east of the Andes.

##### Notes

Galacatos et al. (2004), Barriga (2011).

#### Ageneiosus vittatus

Steindachner 1908

817AA563-5C28-57DF-B7FA-DE1404BBA826

##### Distribution

Middle Orinoco, upper Amazon.

##### Notes

Galacatos et al. (2004) .

#### Auchenipterichthys coracoideus

(Eigenmann & Allen 1942)

B73012DC-6BA9-559E-9AAD-CECABD136A8D

##### Distribution

Upper Amazon.

#### Auchenipterichthys cf. thoracatus

D7A14744-1FDB-5E32-AF30-EE37167774FC

##### Distribution

Amazonia.

##### Notes

Galacatos et al. (2004).

#### Auchenipterus ambyiacus

Fowler 1915

1E456934-9012-506C-8B67-CB6F38C7528F

##### Distribution

Upper and middle Amazon, plus northern drainages.

##### Notes

Galacatos et al. (2004), Guarderas and Jácome-Negrete (2013).

#### Auchenipterus brachyurus

(Cope 1878)

E3A0BAC8-964B-5966-9811-4CCA42EE3DFE

##### Distribution

Upper Amazon.

#### Auchenipterus cf. nuchalis

72FCD48D-CA5E-5B7C-A44D-8C8486DD7E8F

##### Distribution

Widespread Amazon, plus northern drainages.

##### Notes

Barriga (1994).

#### Centromochlus heckelii

(De Filippi 1853)

206E4300-FE77-53F7-8C63-DBE0C2165D46

##### Distribution

Amazon, Orinoco.

##### Notes

Barriga (1994), Galacatos et al. (2004), Barriga (2011).

#### Duringlanis altae

(Fowler 1945)

5C4B79C7-2C7B-528E-A5B7-557D179EDE8B

##### Distribution

South-eastern Colombia, eastern Ecuador.

##### Notes

Barriga (1994).

#### Duringlanis perugiae

(Steindachner 1882)

278A82CC-8398-5E0D-BDD0-A08CB5D9577A

##### Distribution

Upper Amazon.

##### Notes

Barriga (1994).

#### Tatia dunni

(Fowler 1945)

129384E9-C3ED-5FEB-9331-A234A4810636

##### Distribution

South-eastern Colombia, eastern Ecuador, central Brazil.

##### Notes

Galacatos et al. (2004).

#### Tatia intermedia

(Steindachner 1877)

C9513AB9-F104-5298-8711-6E4E42E0D51D

##### Distribution

Amazon, plus northern drainages.

##### Notes

Galacatos et al. (2004), Barriga (2011), Guarderas and Jácome-Negrete (2013).

#### Trachelyopterus galeatus

(Linnaeus 1766)

3240F1A9-5133-5D91-906D-3E6502ED8877

##### Distribution

Amazon, plus northern drainages.

##### Notes

Galacatos et al. (2004), Valdiviezo-Rivera et al. (2018).

#### Tympanopleura atronasus

(Eigenmann & Eigenmann 1888)

E46653B9-3D16-576F-B30B-0B75E473264E

##### Distribution

Middle and upper Amazon.

#### Doradidae

D9136CED-6E22-596D-96D2-0FC2D12ED8B7

#### Acanthodoras spinosissimus

(Eigenmann & Eigenmann 1888)

9E9BC678-06D5-5AB6-9B2B-09BA7067349A

##### Distribution

Amazon, Essequibo.

#### Agamyxis pectinifrons

(Cope 1870)

8DC46A5A-F34C-5F05-B6BB-7DF21E477127

##### Distribution

Amazonia.

##### Notes

Galacatos et al. (2004).

#### Amblydoras affinis

(Kner 1855)

DB40CE7C-E2CF-56C9-8406-0965DD9F29AD

##### Notes

Galacatos et al. (2004).

#### Amblydoras monitor

(Cope 1872)

ECADD65C-A3C1-5A0A-92A3-4D8D637B38FE

##### Distribution

Upper Amazon.

#### Amblydoras nauticus

(Cope 1874)

50A2FEEF-76A6-5BBA-A9FC-996A4CD72568

##### Distribution

Upper Amazon.

#### Anadoras grypus

(Cope 1872)

27844520-DB83-580A-B4FA-190CE2E41E89

##### Distribution

Upper Amazon.

##### Notes

Galacatos et al. (2004).

#### Hemidoras morrisi

Eigenmann 1925

5CF9752A-5F8B-581D-9AFC-DE7B263D8EB9

##### Distribution

Upper Amazon.

#### Leptodoras acipenserinus

(Günther 1868)

D3EF6D4C-A68D-5C9F-A71A-15195BCE5D19

##### Distribution

Upper Amazon of Ecuador, Peru, Brazil.

##### Notes

Barriga (1994).

#### Leptodoras cf. cataniai

7322669E-89FB-5AF8-AC49-0FBB6834EC42

##### Distribution

Napo River Basin, at the locality of Pompeya, sandy island in centre of river.

#### Megalodoras uranoscopus

(Eigenmann & Eigenmann 1888)

07D9B5DA-9357-56B9-B935-7D2CF020AAF8

##### Distribution

Amazon, Essequibo.

#### Nemadoras elongatus

(Boulenger 1898)

C9D42C01-8105-515A-9F4E-857F343A815D

##### Distribution

Amazonia.

#### Nemadoras humeralis

(Kner 1855)

47F59619-2E75-5584-ADF1-23459791B24C

##### Distribution

Amazonia.

##### Notes

Galacatos et al. (2004), Barriga (2011).

#### Ossancora punctata

(Kner 1855)

4D24F7D1-D3E3-5DE3-9AC5-E71968AA143A

##### Distribution

Western, south-western Amazon.

##### Notes

Barriga (1994).

#### Physopyxis ananas

Sousa & Rapp Py-Daniel 2005

326A33E4-881E-55A5-8C63-32617BB8970B

##### Distribution

Amazon, Essequibo.

##### Notes

Galacatos et al. (2004).

#### Platydoras armatulus

(Valenciennes 1840)

14CEA317-2F08-5657-8CC9-F258AF546693

##### Distribution

Widespread Amazon.

##### Notes

Galacatos et al. (2004).

#### Pterodoras granulosus

(Valenciennes 1821)

4C0641FA-0651-54C1-B65E-42434A4B5834

##### Distribution

Amazon, Guianas, Paraná.

#### Tenellus cf. ternetzi

B3D66061-B295-531A-AAD3-7E425BCAE568

##### Distribution

Amazon, Orinoco, Essequibo.

#### Tenellus trimaculatus

(Boulenger 1898)

91BCD401-110B-5477-BC5C-53BA9DC05C8C

##### Distribution

Amazon, Orinoco.

#### Heptapteridae

F018B9DF-C598-54F8-9537-0A5C1326927F

#### Cetopsorhamdia orinoco

Schultz 1944

6FC8E873-107D-5095-AF63-C61585E8546B

##### Distribution

Orinoco River Basin, Ecuador, Colombia and Venezuela.

#### Brachyrhamdia marthae

Sands & Black 1985

A74E6850-8989-5BEC-855C-2B3F75E94175

##### Distribution

Brazil, Ecuador, Bolivia and Peru.

##### Notes

Galacatos et al. (2004).

#### Gladioglanis conquistador

Lundberg, Bornbusch & Mago-Leccia 1991

EEDCE469-D479-5BF5-8F09-B268A901BA71

##### Distribution

Ravine Cotoyacu, downstream from the confluence of the Yasuni River.

##### Notes

Galacatos et al. (2004).

#### Imparfinis longicauda

(Boulenger 1887)

17C5CFDF-6E8E-57FD-9506-80B85355F4F1

##### Distribution

Bobonaza River Basin in upper Partaza River drainage in Ecuador and Rio Corrente in Brazil.

#### Imparfinis nemacheir

(Eigenmann & Fisher 1916)

0D68E74A-90E2-57F5-9074-065C234DE8D5

##### Distribution

Upper Amazon and Orinoco River Basins and Lake Maracaibo Basin: Ecuador, Colombia, Peru and Venezuela.

#### Myoglanis sp. 1

A9EEE5C1-2EB9-508F-99DC-E03153120E96

##### Distribution

Laguna Azul, near the confluence of the Chambira and Tiputini Rivers.

#### Pariolius armillatus

Cope 1872

4902832C-0050-596D-A7F8-35AA4D260064

##### Distribution

Upper Amazon.

##### Notes

Barriga (1994).

#### Pimelodella aff. gracilis

1A274FC5-D3ED-54F5-9871-0411C0427285

##### Distribution

Amazon, Orinoco, La Plata.

#### Pimelodella lateristriga

(Lichtenstein 1823)

47BB065F-FBB3-5596-A213-6608E83DF2DC

##### Distribution

Southern Amazon.

##### Notes

Tobes et al. (2022).

#### Pimelodella sp. 1

21730593-5C7F-52AB-B499-262DFE56F674

##### Distribution

Eastern Ecuador, Tiputini River.

#### Pimelodella cristata

(Müller & Troschel 1849)

D84255F7-E8AA-57C9-805B-504FF4264357

##### Distribution

Amazon and Orinoco River Basins and coastal rivers of north-eastern Brazil (Bolivia, Colombia, Ecuador, French Guiana, Guyana and Peru).

#### Pimelodella cyanostigma

(Cope 1870)

4DF2B5D0-BCC7-5FF6-BB11-1A2744C28DB7

##### Distribution

South America: upper Amazon Basin (Ecuador and Peru).

#### Rhamdia poeyi

Eigenmann & Eigenmann 1888

4A968951-4A1A-5F7C-93AB-25EB5E6FEC81

##### Distribution

Mamoré, upper Napo and Tocantins River Basins: Bolivia, Brazil and Ecuador.

#### Rhamdia quelen

(Quoy & Gaimard 1824)

A642B764-4138-5EA0-9C4F-2AA2F29B336A

##### Distribution

Mexico to Argentina.

#### Pimelodidae

06039065-FE47-5673-BD20-A18AC5131523

#### Aguarunichthys torosus

Stewart 1986

0444281E-989F-5018-B2D0-06F396762825

##### Distribution

Amazon River drainage (Ecuador, Peru and Bolivia).

#### Brachyplatystoma filamentosum

(Lichtenstein 1819)

1A083AAD-2114-5275-9DBB-D693D6D4BB11

##### Distribution

Amazon, plus northern drainages.

##### Notes

Barriga (1994).

#### Brachyplatystoma juruense

(Boulenger 1898)

F1867246-0E08-521A-A222-744E6781E9A7

##### Distribution

Amazon, Orinoco.

#### Brachyplatystoma platynema

Boulenger 1898

FF50E00F-D6F6-54DF-8533-D89BB9CF4125

##### Distribution

Amazon, Orinoco.

#### Brachyplatystoma tigrinum

(Britski 1981)

2D2211A2-295A-5E09-A937-61426968B23D

##### Distribution

Amazon.

#### Calophysus macropterus

(Lichtenstein 1819)

096AC8FA-D4C7-532D-B9BE-76423B97973B

##### Distribution

Amazon, Orinoco.

##### Notes

Barriga (1994), Galacatos et al. (2004), WCS (2007), Jácome-Negrete (2013), Jácome-Negrete et al. (2018).

#### Cheirocerus eques

Eigenmann 1917

4A3B002E-62A1-5132-98BA-ABE36755D30F

##### Distribution

Amazon.

##### Notes

Barriga (1994).

#### Cheirocerus goeldii

(Steindachner 1908)

0F5483F9-0F2F-5B69-8374-08A37ADBC2F6

##### Distribution

South-western Amazonia.

#### Hemisorubim platyrhynchos

(Valenciennes 1840)

74CFB169-06F9-5FB1-BCAE-6D47CB46E162

##### Distribution

Widespread Amazon.

##### Notes

Barriga (1994), Galacatos et al. (2004).

#### Hypophthalmus edentatus

Spix & Agassiz 1829

5EB5B24B-901C-58D1-A4FE-04359D218FBF

##### Distribution

Amazon, plus northern drainages.

##### Notes

Jácome-Negrete (2013), Jácome-Negrete et al. (2018).

#### Megalonema platycephalum

Eigenmann 1912

5DFD4FBF-8263-5B50-A9FD-6E4DCD68CDA8

##### Distribution

Amazon, Orinoco, Essequibo.

#### Phractocephalus hemioliopterus

(Bloch & Schneider 1801)

2EBBEE22-963E-5215-A6A1-A6E9B0A9B170

##### Distribution

Widespread Amazon.

#### Pimelodus altissimus

Eigenmann & Pearson 1942

4FD61021-4BFD-5BDF-9AD1-D791BCD50F20

##### Distribution

Amazon.

#### Pimelodus blochii

Valenciennes 1840

350A087E-8B58-5DAB-9B61-C5CC53B3CE83

##### Distribution

Amazon, Orinoco, Essequibo.

##### Notes

Jácome-Negrete (2013), Jácome-Negrete et al. (2018).

#### Pimelodus jivaro

Eigenmann & Pearson 1942

C965D5AC-7729-506D-BEEA-6B61852A6166

##### Distribution

Upper Amazon of Ecuador, Peru.

#### Pimelodus ornatus

Kner 1858

D03EC88E-B144-5A60-BA54-F892CEF7F5A6

##### Distribution

Amazon, plus northern drainages, Paraná.

##### Notes

Barriga (1994).

#### Pimelodus pictus

Steindachner 1876

E08877BA-8E8D-54AC-A0FB-964D7561DE86

##### Distribution

Amazon, Orinoco.

##### Notes

Barriga (1994).

#### Pimelodus sp. 1

32A71FA1-E509-5F3F-8758-C6AE0937E86F

##### Distribution

Napo River, next to Tiputini military detachment.

#### Pimelodus sp. 2

05737743-F2FF-5E03-B75F-D3840025A805

##### Distribution

Napo River, Anangucocha Lagoon.

#### Pinirampus pirinampu

(Spix & Agassiz 1829)

B497CB92-BC52-5D99-A945-2FB5496ACBCE

##### Distribution

Amazon, Orinoco, Essequibo, Paraná.

##### Notes

Barriga (1994), Galacatos et al. (2004), Jácome-Negrete (2013), Jácome-Negrete et al. (2018).

#### Platystomatichthys sturio

(Kner 1858)

4621515E-E608-5998-959E-682C6C9E8A42

##### Distribution

Amazon.

##### Notes

Barriga (1994), Galacatos et al. (2004).

#### Pseudoplatystoma fasciatum

(Linnaeus 1766)

A714C985-E114-5F7C-AD8C-967E956F19FB

##### Distribution

Amazon, Orinoco, Essequibo, Paraná.

##### Notes

Barriga (1994), Jácome-Negrete (2013).

#### Pseudoplatystoma punctifer

(Castelnau 1855)

43331173-E2F9-50CA-8A0D-157A2D4D44D8

##### Distribution

Amazon.

##### Notes

Jácome-Negrete et al. (2018).

#### Pseudoplatystoma tigrinum

(Valenciennes 1840)

85D45C29-D6A6-5DC7-8024-015FA49B975B

##### Distribution

Amazon, Orinoco.

##### Notes

Barriga (1994), Jácome-Negrete (2013), Jácome-Negrete et al. (2018).

#### Sorubim elongatus

Littmann, Burr, Schmidt & Isern 2001

2CFD78CD-4987-52B2-A579-81B4A58F511B

##### Distribution

Amazon, Orinoco, Essequibo.

##### Notes

Galacatos et al. (2004).

#### Sorubim lima

(Bloch & Schneider 1801)

B8E6EC52-D10F-510C-8FB4-0236F7F73236

##### Distribution

Amazon, Orinoco, Paraná.

##### Notes

Barriga (1994), Galacatos et al. (2004).

#### Sorubim maniradii

Littmann, Burr & Buitrago-Suárez 2001

787E2F7C-947A-59C7-A246-1DDB3705D2A0

##### Distribution

Middle, upper Amazon.

#### Zungaro zungaro

(Humboldt 1821)

75C7313E-7E81-511A-BDEA-5A97C29072AD

##### Distribution

Amazon, Orinoco.

##### Notes

Barriga (1994).

#### Pseudopimelodidae

3B6740AD-6C54-57B3-989F-38FA0B8E4D1C

#### Batrochoglanis villosus

(Eigenmann 1912)

86CBFD8C-2183-581C-9FC5-C227E17E800F

##### Distribution

Amazon, Orinoco, Essequibo.

#### Microglanis pellopterygius

Mees 1978

B4008389-C6EA-5CDC-895A-A4D545E26255

##### Distribution

Eastern Ecuador.

##### Notes

Barriga (2011).

#### Synbranchiformes

15AB386D-60C4-550B-ABED-079409E12762

#### Synbranchidae

63FA5B15-7E35-54B5-899F-632C5CDBBBC0

#### Synbranchus marmoratus

Bloch 1795

31EC3166-7377-5612-B863-3899B393CF70

##### Distribution

Mexico to northern Argentina

##### Notes

Barriga (1994), Guarderas and Jácome-Negrete (2013).

#### Carangiformes

09994CC2-1883-56DF-BCF4-D6F5D3C97DCD

#### Achiridae

904315BF-796B-50F2-87CE-A8627B428F71

#### Apionichthys menezesi

Ramos 2003

CD4DF60D-D9F7-5CBB-9FE8-123A8BA16E22

##### Distribution

Rio Napo in Ecuador, Rio Negro, Orinoco, Amazon.

##### Notes

Barriga (2011), Valdiviezo-Rivera et al. (2018).

#### Apionichthys nattereri

(Steindachner 1876)

2CE14492-F90B-5F58-8C55-A2E47F07F117

##### Distribution

Amazon.

#### Hypoclinemus mentalis

(Günther 1862)

38AFBE9C-64B0-5775-8207-B1F81F65BEAC

##### Distribution

Amazon, Orinoco, Essequibo

##### Notes

Barriga (1994), Galacatos et al. (2004), Guarderas and Jácome-Negrete (2013), Jácome-Negrete (2013), Jácome-Negrete et al. (2018).

#### Cichlidae

C5536AC0-B1CB-5418-A15F-CC5CBEB319F3

#### Cichliformes

4AD763CD-9035-5E23-9BBB-CAC96FD78B24

#### Aequidens cf. diadema

65F8A021-C186-58ED-8272-2D02073094D1

##### Distribution

Amazon, Orinoco, Negro.

##### Notes

Galacatos et al. (2004).

#### Aequidens tetramerus

(Heckel 1840)

1B369400-1E7E-5D4A-88EA-3DF9E4B28D1A

##### Distribution

Western Amazonia, Orinoco, plus northern drainages.

##### Notes

Galacatos et al. (2004), Guarderas and Jácome-Negrete (2013), Valdiviezo-Rivera et al. (2018), Tobes et al. (2022).

#### Apistogramma amoena

Cope, 1872

90446E7F-B515-5D8E-A4D5-95B17309FAD3

##### Distribution

Eastern Ecuador, north-eastern Peru.

##### Notes

Considered a species inquirenda in Apistogramma (*sensu* Kullander (2003)).

#### Apistogramma cruzi

Kullander 1986

CC823097-35F4-5426-94A9-76182DCC15B3

##### Distribution

Peru, Ecuador, Colombia.

##### Notes

Barriga (1994), Galacatos et al. (2004), Barriga (2011).

#### Apistogramma payaminonis

Kullander 1986

242483D2-D2DE-51FC-B630-2BEEEBB9BBA7

##### Distribution

Napo Basin in Ecuador.

#### Apistogramma playayacu

Römer, Beninde & Hahn 2011

A6BBD38F-2F9A-5C4A-835D-C89E39BDC7A7

##### Distribution

Napo Basin in Ecuador.

#### Apistogramma sp. 1

A1278325-5A74-5373-A743-154CC388CCF9

##### Distribution

Eastern Ecuador, Jatuncocha Lake.

#### Astronotus ocellatus

(Agassiz 1831)

5AAE1CBF-25DE-5789-8AB2-7518F746EE57

##### Distribution

Western Amazonia, Orinoco.

##### Notes

Barriga (1994), Galacatos et al. (2004), Guarderas and Jácome-Negrete (2013), Jácome-Negrete (2013), Valdiviezo-Rivera et al. (2018).

#### Bujurquina mariae

(Eigenmann 1922)

B4D868DB-FA49-5915-B3A7-EA9FDE310D2F

##### Distribution

South America: Meta River Basin of the Orinoco River Basin, Colombia.

#### Bujurquina moriorum

Kullander 1986

DFF8DB1B-19C6-5583-BD92-C8F08E66CA19

##### Distribution

Napo Basin in Ecuador.

##### Notes

Barriga (1994), Barriga (2011).

#### Bujurquina syspilus

(Cope 1872)

6C0AC859-F79D-50C9-A035-8A87F85C3185

##### Distribution

Amazon tributaries in Peru, Ecuador.

#### Caquetaia myersi

(Schultz 1944)

235497FF-5052-585F-830E-55B6F207CB68

##### Distribution

Eastern Ecuador.

##### Notes

Barriga (1994), Barriga (2011).

#### Chaetobranchus flavescens

Heckel 1840

BC7B88A7-698F-5F60-A1B2-55CF55127A13

##### Distribution

Amazon, plus northern drainages, Orinoco.

##### Notes

Barriga (1994), Barriga (2011).

#### Cichla monoculus

Spix & Agassiz 1831

1B5BEE9C-4EAE-5903-A82E-F447A89D8BA4

##### Distribution

Western Amazonia.

##### Notes

Barriga (1994), Galacatos et al. (2004), Guarderas and Jácome-Negrete (2013), Jácome-Negrete (2013), Jácome-Negrete et al. (2018).

#### Cichlasoma cf. amazonarum

878A171B-7D7A-5CBD-9DD8-588A8B7B2D61

##### Distribution

Western Amazonia, plus northern drainages.

##### Notes

Barriga (2011).

#### Crenicara punctulata

(Günther 1863)

A659E773-237F-590E-BD80-FBED62482A66

##### Distribution

Peru, Ecuador, Colombia, Brazil, Essequibo.

##### Notes

Barriga (1994), Guarderas and Jácome-Negrete (2013).

#### Crenicichla sedentaria

Kullander 1986

E7F86915-4E4A-5924-B241-23D242865BC6

##### Distribution

Upper Amazon of Ecuador, Peru.

#### Heroina isonycterina

Kullander 1996

D59E8D1F-58D0-51F7-8D73-5C3A2EEA63F7

##### Distribution

Upper Amazon of Ecuador, Colombia.

#### Heros efasciatus

Heckel 1840

94413241-1AE2-571B-8908-1F3D11B2E705

##### Distribution

Western Amazonia, Ecuador, Peru, Brazil.

##### Notes

Barriga (2011), Barriga (1994), Galacatos et al. (2004).

#### Hypselecara temporalis

(Günther 1862)

218BB315-7E58-5726-8043-F340D18F8704

##### Distribution

Western Amazonia, Colombia, Ecuador, Peru, Brazil.

##### Notes

Barriga (1994), Galacatos et al. (2004), Guarderas and Jácome-Negrete (2013).

#### Laetacara flavilabris

(Cope 1870)

EB5D36A3-B1B4-5CF3-8098-2FFFEDF4F363

##### Distribution

Western Amazonia, Ecuador, Peru, Brazil.

##### Notes

Barriga (1994), Galacatos et al. (2004).

#### Lugubria cincta

(Regan 1905)

7314174E-53A2-5F26-90E0-23CA01D923D0

##### Distribution

Napo, Amazon.

##### Notes

Galacatos et al. (2004), Valdiviezo-Rivera et al. (2018), Tobes et al. (2022).

#### Lugubria johanna

(Heckel 1840)

DAB712AD-9B12-5B11-98B5-E5240D6C0CBE

##### Distribution

Amazon, plus northern drainages.

#### Mesonauta cf. insignis

CB84C596-611F-5A27-A708-C9732637332D

##### Distribution

Amazon River and Orinoco River Basins (Bolivia, Brazil, Colombia, Ecuador, Peru and Venezuela).

##### Notes

Barriga (1994).

#### Mesonauta mirificus

Kullander & Silfvergrip 1991

CF3D74B4-4C57-5B34-A57B-5ECDE0B6B836

##### Distribution

Western Amazonia, Colombia, Ecuador, Peru.

##### Notes

Barriga (2011).

#### Satanoperca jurupari

(Heckel 1840)

497D0B3F-F168-5333-959C-46D65A158352

##### Distribution

Widespread Amazonia, Colombia, Ecuador, Peru, Bolivia, Orinoco.

##### Notes

Galacatos et al. (2004), Guarderas and Jácome-Negrete (2013), Jácome-Negrete (2013), Jácome-Negrete et al. (2018).

#### Saxatilia anthurus

(Cope 1872)

F748F5A6-91C1-520E-9674-571E455ADED8

##### Distribution

Upper Amazon of Ecuador, Peru.

##### Notes

Galacatos et al. (2004).

#### Saxatilia lucius

(Cope 1870)

C9EBA4D8-0CA8-5CAD-AA8A-F2E1BCEE0B97

##### Distribution

Upper Amazon of Ecuador, Peru.

#### Saxatilia proteus

(Cope 1872)

6EE1CCA9-1F57-54BB-B438-8DA589BCFADD

##### Distribution

Upper Amazon of Ecuador, Peru.

##### Notes

Barriga (1994), Galacatos et al. (2004).

#### Cyprinodontiformes

CE3EA976-8321-5341-A2B8-38FE89565649

#### Rivulidae

0834B6B7-B898-5B11-9F82-419AF3A3356D

#### Anablepsoides erberi

(Berkenkamp 1989)

95C84EE3-DF00-532E-8F7F-6965D483669A

##### Distribution

Eastern Ecuador.

#### Anablepsoides limoncochae

(Hoedeman 1962)

760DAFAE-A75D-5B1F-89E2-EC32D3E017D2

##### Distribution

Eastern Ecuador.

##### Notes

Galacatos et al. (2004).

#### Anablepsoides urophthalmus

(Günther 1866)

F088D3F7-44D2-5803-A430-CF86BEF45638

##### Distribution

Amazon.

##### Notes

Barriga (1994), Barriga (2011).

#### Beloniformes

53AF61E2-6BF8-5593-8739-1844216BC676

#### Belonidae

0CDFC095-9FB3-5B97-ADBF-4AB9F1E78D7D

#### Potamorrhaphis guianensis

(Jardine 1843)

D913AC4E-6999-55A6-A7DC-1C91EDB53A89

##### Distribution

Amazon, Orinoco, Guianas.

##### Notes

Barriga (1994), Barriga (2011).

#### Pseudotylosurus angusticeps

(Günther 1866)

14199F0E-8243-5AED-BEBB-9C85EA45CFAD

##### Distribution

Eastern Ecuador, eastern Peru, Paraguay, Paraná.

##### Notes

Barriga (1994).

#### Pseudotylosurus microps

(Günther 1866)

EA2EFBF3-2D90-51BE-9262-3CA86AFEDD48

##### Distribution

Amazon, Orinoco, Guianas.

##### Notes

Guarderas and Jácome-Negrete (2013).

#### Acanthuriformes

35135C71-661E-53B9-8D64-AC47593C144F

#### Sciaenidae

795ABA35-B5B6-585E-8DF2-D2A80F4104A3

#### Pachyurus stewarti

Casatti & Chao 2002

C2D60AE7-E1B8-575E-87CD-CE0BAB3329F0

##### Distribution

Eastern Ecuador.

##### Notes

Barriga (2011).

#### Plagioscion squamosissimus

(Heckel 1840)

38068855-338C-57EF-931F-8836754B9948

##### Distribution

Amazon, plus northern drainages, Paraguay, Paraná.

##### Notes

Barriga (1994), Galacatos et al. (2004), WCS (2007), Barriga (2011), Guarderas and Jácome-Negrete (2013), Jácome-Negrete (2013), Valdiviezo-Rivera et al. (2018), Jácome-Negrete et al. (2018).

#### Tetraodontiformes

D6B77281-E322-5098-B230-3DFC1EE22B85

#### Tetraodontidae

2132BC0A-82B6-5FCF-9C6F-653A35787A89

#### Sphoeroides asellus

(Müller & Troschel 1849)

2DED85FB-5461-549F-896A-30D9E97CEE81

##### Distribution

Amazon, Orinoco, Essequibo.

##### Notes

Barriga (1994), Galacatos et al. (2004), Guarderas and Jácome-Negrete (2013), Jácome-Negrete et al. (2018).

## Analysis

**Figure 3.**
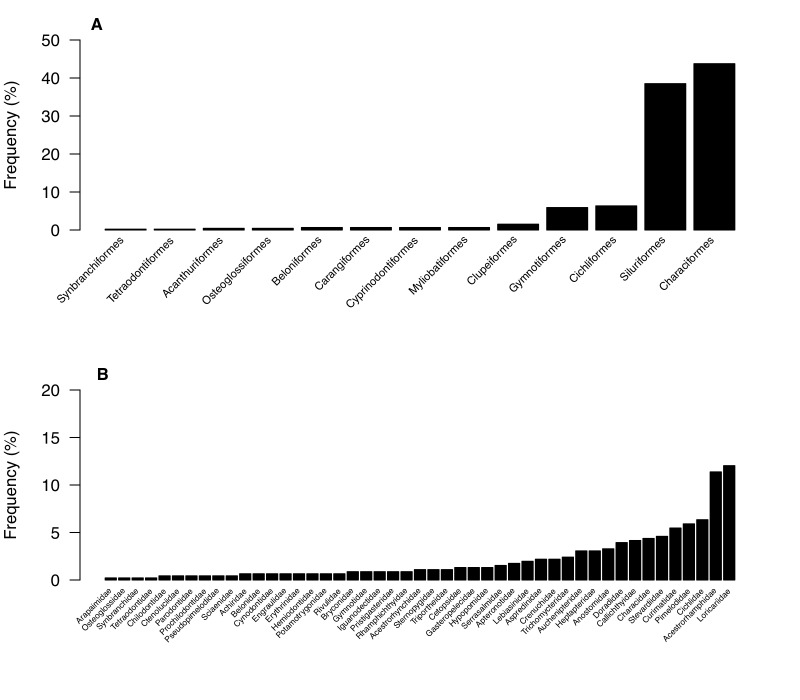
Species richness by order (A) and family (B), where frequency represents the proportion of unique species from each order or family in the database.

**Figure 4.**
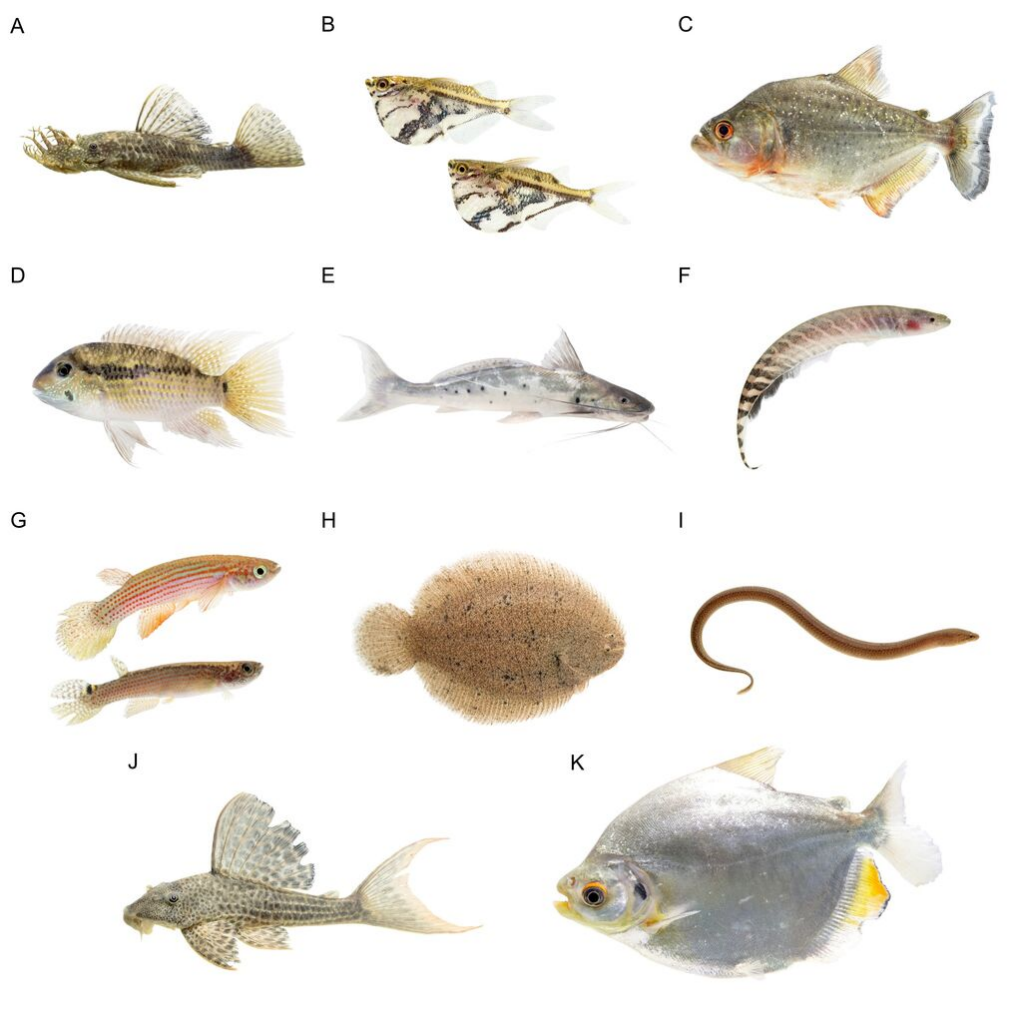
Examples of species from Yasuní National Park denoting their morphological and phylogenetic diversity. **A** *Ancistrusmalacops* (Loricariidae); **B** *Carnegiellastrigata* (Gasteropelecidae); **C** *Serrasalmusrhombeus* (Serrasalmidae); **D** *Bujurquinamoriorum* (Cichlidae); **E** *Calophysusmacropterus* (Pimelodidae); **F** *Gymnotuscarapo* (Gymnotidae); **G** *Anablepsoideslimoncochae* (Rivulidae); **H** *Hypoclinemusmentalis* (Achiridae); **I** *Synbranchusmarmoratus* (Synbranchidae); **J** *Hypostomushemicochliodon* (Loricariidae); **K** *Mylossomaalbiscopum* (Serrasalmidae). Photos: courtesy of José Vieira (https://www.ex-situphotography.com/).

Fish species in YNP span 13 orders, 47 families, 239 genera and 458 species (Fig. 3). Characiformes and Siluriformes contributed the most to species richness (43% and 38%, respectively). These are followed by Cichliformes and Gymnotiformes. The richest families in YNP are Loricariidae and Acestrorhamphidae (Figs 3, 4). Additionally, 23 families each account for at least 1% of the total fish richness (Fig. 3).

The updated list more than doubles the previous YNP inventory (Barriga 1994) and accounts for 70% of the known fish species in the Napo Basin and 21.9% of the entire Amazon Basin (Jézéquel et al. 2020). The fish richness in YNP surpasses that of other Amazonian reserves of comparable or larger size (Table 1). This underscores Yasuní as a biodiversity hotspot specifically for fishes, which represent the second most species-rich vertebrate group in YNP after birds (Bass et al. 2010).

## Discussion

Our study added 205 species to the 253 previously listed by Barriga (1994) for the YNP, representing an 80% increase in species richness. This substantial increase is primarily attributed to a significant expansion of the sampling area, encompassing zones that were not included in the Barriga (1994) list. These additional data were obtained through collection campaigns conducted by MEPN (e.g. Barriga (2011)), samplings in the Cononaco and Curaray Rivers in 2014, unpublished data), MECN (Valdiviezo-Rivera et al. 2018) and USFQ (Swing 2017). Other contributing factors include the description of new species in the YNP (Provenzano-Rizzi and Barriga-Salazar 2020, Provenzano-Rizzi et al. 2024), new records (Chuctaya et al. 2021, Chuctaya et al. 2023), increased collections from environmental consulting activities, taxonomic re-identifications and curatorial activities involving the biological collections analysed in the present study.

Our results also align with the common structure of the Neotropical ichthyofauna, characterised by the dominance of ostariophysan fishes (Sleen and Albert 2018). Characiform species showed the highest percentage, followed by Siluriformes. This proportional dominance at the order level has also been observed in other inventories conducted in areas surrounding the YNP, such as the Upper Napo River Basin (Anaguano-Yancha and Cueva 2016), Cuyabeno (Barriga et al. 2016) and the Pastaza River Basin (Rivadeneira et al. 2010). Similar patterns have been reported in other regions of the western (Meza-Vargas et al. 2021), central (Oliveira et al. 2020) and lower Amazon (Fróis et al. 2021).

This pattern extends to the family level, with Loricariidae (55 species) and Acestrorhamphidae (52 species) being the most species-rich families. Loricariidae has long been amongst the most diverse families, historically ranking second in species richness (Roxo et al. 2019). However, taxonomic revisions splitting Acestrorhamphidae, Characidae and Stevardiidae from the broader Characidae (Melo et al. 2024) have elevated Loricariidae to the top position. The high species richness of Loricariidae reflects significant lineage and ecological diversification (Lujan et al. 2012), whereas the diversity of characiform families, widely distributed across the Neotropics (Reis et al. 2003, Albert and Reis 2011), results from habitat shifts, morphological diversification and trophic niche adaptations (Guisande et al. 2012). Furthermore, high relative diversity of Cichlidae (29) and Pimelodidae (28) coincides with diversity patterns described in the Upper Amazon (Dagosta and Pinna 2019, Meza-Vargas et al. 2021). Although these patterns in the YNP coincide with others previously recorded in the region, a comparative analysis between its extension and the recorded species shows a greater concentration of diversity per area in relation to other historically diverse protected areas, such as Mamirauá Sustainable Development Reserve with 34% more species or even with areas that quadruple its extension, such as Tumucumaque National Park registering 120% greater fish richness (Table 1).

The extraordinary fish diversity in YNP results from multiple ecological and biogeographic factors. First, the convergence of white- and black-water rivers in the YNP support distinct fish communities typical of sediment-rich white waters and tannin-rich black waters (Bogotá-Gregory et al. 2020). This area has been categorised as one of the ecoregions with the highest density of fish species in the Neotropics (Albert and Reis 2011). Second, major rivers in this area have been historically shaped by shifting connections and isolations fostering diversification of fish assemblages (Albert et al. 2006, Londoño-Burbano and Britto 2023). Finally, the YNP’s connection to central Amazonia likely facilitates extensive sympatric diversification in the western Amazon (Wesselingh and Carina 2011, Londoño-Burbano and Britto 2023). Remarkably, the updated list for YNP reports no introduced species, highlighting it as one of Ecuador’s few protected areas where native fish populations remain unthreatened by the impacts of exotic species (Aguirre et al. 2021). This underscores the importance of conservation efforts in YNP, which provide ecosystem services such as maintenance of biodiversity and provision of food security (Berlin and Markell 1977, Dufour 1983, Sirén 2011).

**Figure 5.**
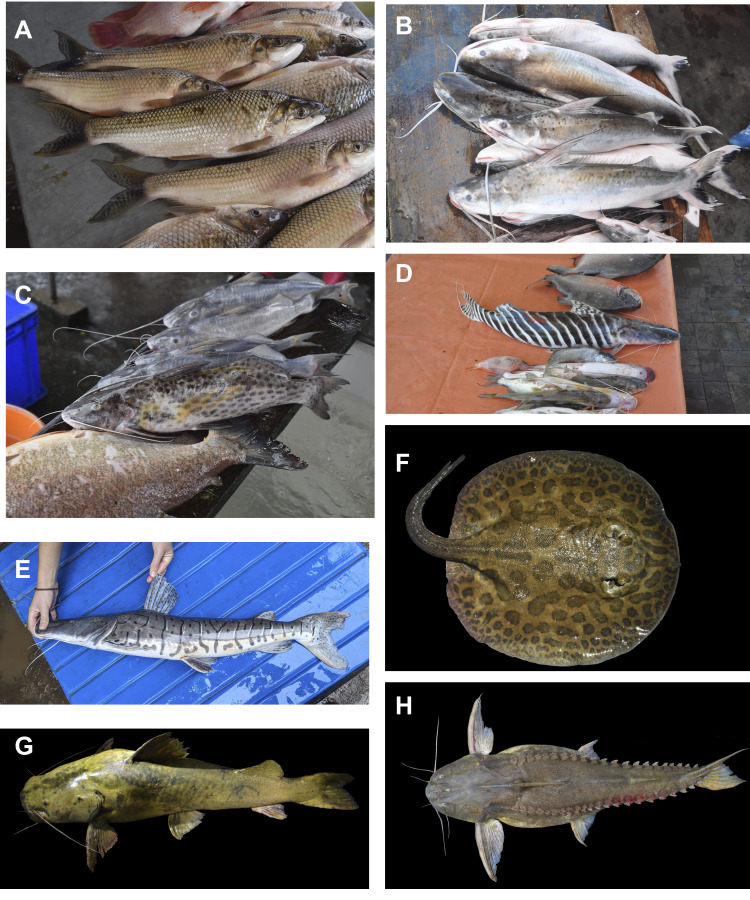
Examples of species exploited in artisanal fisheries for livelihoods and food security in Yasuní National Park. **A** *Prochilodusnigricans* (Prochilodontidae); **B** *Platynematichthysnotatus* (Pimelodidae); **C** *Aguarunichthystorosus* (Pimelodidae); **D** *Brachyplatystomatigrinum* (Pimelodidae); **E** *Pseudoplatystomafasciatum* (Pimelodidae); **F** *Potamotrygonmotoro* (Potamotrygonidae); **G** *Zungarozungaro* (Pimelodidae); **H** *Pterodorasgranulosus* (Doradidae). Photos: courtesy of Kelly Swing (A, B, C and D), Jonathan Valdiviezo-Rivera (F, G and H) and Daniel Escobar-Camacho (E).

The Park’s fish diversity reflects a wide range of ecologies, morphologies and life history traits (Fig. 4). Indigenous communities depend on at least 72 species for consumption, trade and medicinal uses, with large predatory pimelodids being particularly favoured (García-Dávila et al. 2013, García-Dávila et al. 2018, Duponchelle 2021). Conservation concerns include 19 threatened species in YNP, many of which are heavily exploited by artisanal fisheries (Fig. 5) (Escobar-Camacho 2024).

**Figure 6.**
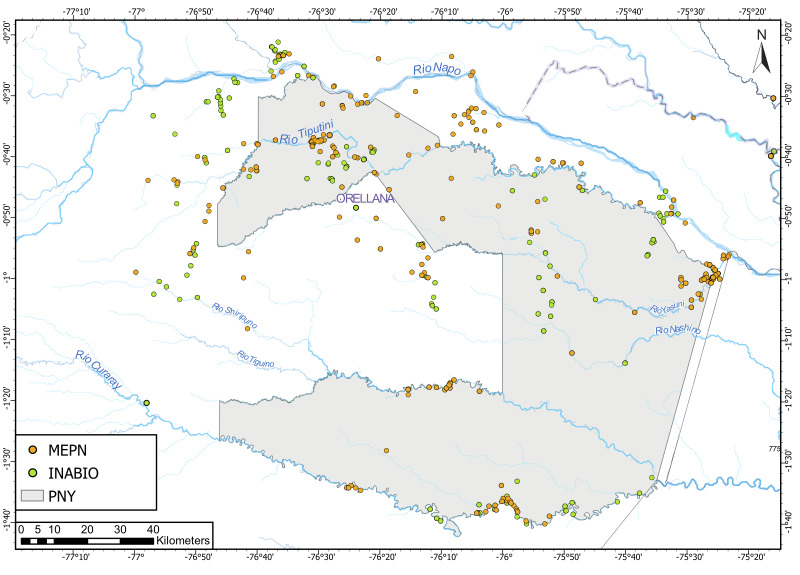
Yasuní National Park and sampling sites based on records from MEPN and MECN-DP.

Most fish collections from MEPN and MECN-DP (Fig. 6) are concentrated in medium to large rivers such as the Napo, Tiputini, Yasuní and Shiripuno, leaving the southern interior of YNP under-sampled. Given the high ichthyological diversity across rivers, forest streams, and lagoons of the northern area of YNP (Ibarra and Stewart 1989, Bojsen and Barriga 2002, Galacatos et al. 2004), identifying these sampling gaps is crucial for understanding migration, reproduction and feeding patterns (Duponchelle 2021). Expanding surveys in these areas may document species recorded outside YNP or refine distribution knowledge of species already present. This has been evident in studies of northern YNP, where cryptic diversity and rediscovered species have been reported (Escobar-Camacho et al. 2015, Provenzano-Rizzi and Barriga-Salazar 2020, Valdiviezo-Rivera et al. 2021, Říčan et al. 2023).

Beyond geographic sampling limitations, species lists for large regions like the western Amazon are further complicated by cryptic diversity,rare species and behavioural adaptations. Small-bodied or elusive species often evade detection, requiring targeted efforts for confirmation (Swing et al. 2014). Some species are rarely captured due to habitat preferences or migration patterns, as seen in recent rediscoveries like *Rhyacoglanispulcher* and *Micromyzonakamai* in the Curaray River Basin (Chuctaya et al. 2021, Chuctaya et al. 2023). Large species also present sampling challenges, though evidence, such as drifting larvae of large pimelodids in the Napo River (Hermann et al. 2021), confirms their presence. Similarly, migratory siluriforms such as *Aguarunichthystorosus* and *Pterodorasgranulosus* (Fig. 5), while absent from museum collections, frequently appear in nearby markets, indicating their likely presence in the study area.

The intent of this list is to provide a baseline for future studies that would use this information to develop hypotheses and better understand fish diversity in the Ecuadorian Amazon. This list is also intended to provide basic information to the scientific community and society at large so that adequate measures may be applied for the conservation of these natural resources and the maintenance of genetic diversity.

### Conclusions

By compiling curated datasets, we developed an updated list of freshwater fish species in YNP, doubling species richness compared to previous lists. This list represents 70% of the Napo River Basin’s known species and 20% of the Amazon Basin’s. Our results highlight the ecological and cultural importance of YNP, including its provision of services to indigenous communities and the threats several species face from anthropogenic disturbances. We identify under-sampled habitats requiring further study and underscore the need for preserving YNP’s biodiversity and ecosystem services. This list aims to support future research, management and conservation efforts.

## Figures and Tables

**Figure 2.**
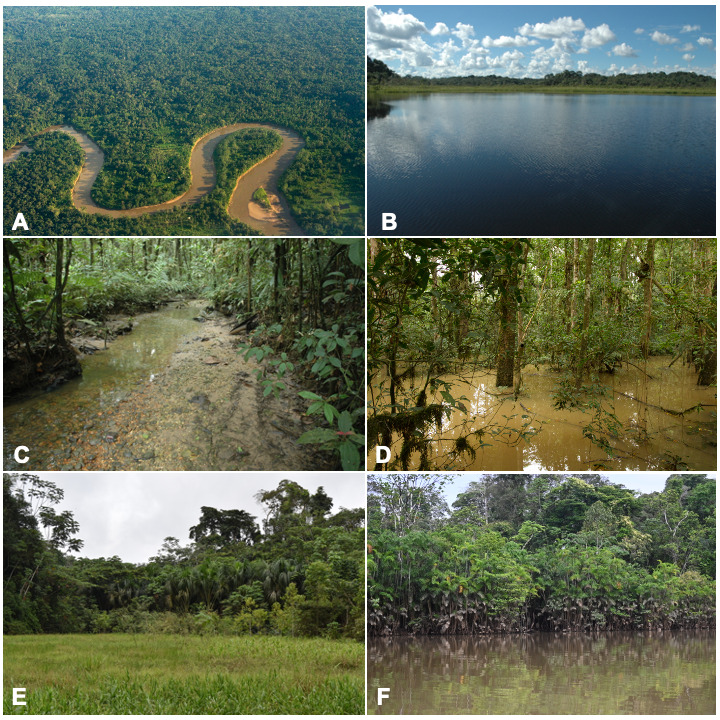
Different water types and ecosystems within YNP. **A** White waters of the Tiputini River (Evergreen Lowland Forest of the Napo-Curaray); **B** Black waters of Lake Añangu (Evergreen Lowland Forest of the Aguarico-Putumayo-Caquetá); **C** Clear water stream (Tiputini River Basin); **D** Flooded forest in the rainy season (Tiputini River Basin); **E** Marsh (Flooded Grassland of the Amazon fluvial plains); **F** Temporary lake of the Flooded Palm Forests, Amazon alluvial plains. Photos: courtesy of Esteban Suárez (A and D) and Kelly Swing (B, C, E and F).

**Table 1.** Fish richness from different protected areas of the Amazon Basin.

**Protected Area**	**Area (km²)**	# **Species**	**Reference**
Yasuní National Park (YNP)	9,823	458	This study
Mamirauá Sustainable Development Reserve (MSDR), Brazil	11,137	340	Queiroz and Peralta (2010)
Tumucumaque National Park, Brazil	38,867	207	Bernard (2008)
Jau National Park, Brazil	22,720	320	IUCN (2000)
Madidi National Park, Bolivia	18,958	333	National-Parks (2022)
Manu National Park, Peru	17,162.95	201	Parques-Nacionales (2019)
La Paya National Natural Park, Colombia	4,401	230	Peña Alzáte et al. (2020)
